# Injectable Thixotropic β–Cyclodextrin–Functionalized Hydrogels Based on Guanosine Quartet Assembly

**DOI:** 10.3390/ijms22179179

**Published:** 2021-08-25

**Authors:** Monica-Cornelia Sardaru, Irina Rosca, Simona Morariu, Elena-Laura Ursu, Razvan Ghiarasim, Alexandru Rotaru

**Affiliations:** 1 Centre of Advanced Research in Bionanoconjugates and Biopolymers, “Petru Poni” Institute of Macromolecular Chemistry, Romanian Academy, Grigore Ghica Voda Alley 41 A, Iasi 700487, Romania; sardaru.monica@icmpp.ro (M.-C.S.); rosca.irina@icmpp.ro (I.R.); ursu.laura@icmpp.ro (E.-L.U.); ghiarasim.razvan@icmpp.ro (R.G.); 2 Natural Polymers, Bioactive and Biocompatible Materials, “Petru Poni” Institute of Macromolecular Chemistry, Romanian Academy, Grigore Ghica Voda Alley 41 A, Iasi 700487, Romania; smorariu@icmpp.ro

**Keywords:** supramolecular hydrogel, guanosine quartet, β–cyclodextrin, injectable hydrogel, antimicrobial activity

## Abstract

Facile method for the preparation of β–cyclodextrin–functionalized hydrogels based on guanosine quartet assembly was described. A series of seven hydrogels were prepared by linking β–cyclodextrin molecules with guanosine moieties in different ratios through benzene–1,4–diboronic acid linker in the presence of potassium hydroxide. The potassium ions acted as a reticulation agent by forming guanosine quartets, leading to the formation of self–sustained transparent hydrogels. The ratios of the β–cyclodextrin and guanosine components have a significant effect on the internal structuration of the components and, correspondingly, on the mechanical properties of the final gels, offering a tunablity of the system by varying the components ratio. The insights into the hydrogels’ structuration were achieved by circular dichroism, scanning electron microscopy, atomic force microscopy, and X–ray diffraction. Rheological measurements revealed self–healing and thixotropic properties of all the investigated samples, which, in combination with available cyclodextrin cavities for active components loading, make them remarkable candidates for specific applications in biomedical and pharmaceutical fields. Moreover, all the prepared samples displayed selective antimicrobial properties against *S. aureus* in planktonic and biofilm phase, the activity also depending on the guanosine and cyclodextrin ratio within the hydrogel structure.

## 1. Introduction

Hydrogels represent complex three–dimensional cross–linked networks that can retain large amounts of water while preserving their structure [1,2,3]. The high water content and, in certain cases, good biocompatibility have contributed to the applications of hydrogels in biomedically related fields as drug delivery systems or tissue engineering [3,4,5,6,7]. The crosslinking strategy chosen during the design of the hydrogel structure strongly influences the properties of the final material. Typically, the covalent crosslinking yields robust and highly stable elastomeric hydrogels, while hydrogels with non–covalent physical crosslinking such as hydrogen bonding [8,9,10], electrostatic interactions [11,12], or host–guest recognition [13,14], also known as supramolecular hydrogels, produce less stable materials but with unique properties. Self–healing behavior is among the most important properties of supramolecular hydrogels that has determined their applications in a variety of fields, including targeted drug and gene delivery [15,16,17,18], tissue engineering [17,19,20], and wound dressings [21,22,23]. Additionally, the composition of the hydrogels structural units is particularly important in the view of active substances loading. In this regard, cyclodextrin–based supramolecular hydrogels are of a particular interest since they permit successful filling of poor soluble compounds through host–guest interaction, leading to enhanced solubility of the substance [24,25]. Hence, the search for the strategies in which the cyclodextrin moiety is not involved in the structure of the hydrogel network as reticulation agent through multiple host–guest interactions [6,14] and thus can fully display its complexation capabilities, is of a particular interest. Over the past decade, a number of topical reports on successful strategies for the insertion of cyclodextrin molecules as a drug loading unit inside the hydrogel has been reported [26,27,28]. Analyzing the existing data, we observed that the usual strategy to insert the cyclodextrin molecules into the hydrogel structure typically involves its grafting onto the polymer chain of choice. To the best of our knowledge, no data on preparation of cyclodextrin–based hydrogels by controlled self–assembly of small molecules have been reported thus far.

In our previous works, we have reported the preparation of guanosine–based supramolecular hydrogels with high water content as scaffolds for cell growing applications [29,30]. Water soluble guanosine analogues are known to form hydrogels by the metal–assisted formation of guanosine quartets (G4) as the gelation triggers [31,32,33]. Here, we report on the preparation of β–cyclodextrin (β–CD) hydrogel with tunable properties by its covalent insertion into the G4s network of the hydrogel. CD molecules are cyclic oligosaccharides consisting of six or more units of glucopyranoside, which, due to specific interaction of organic molecules with cyclodextrins hydrophobic cavities, have widely been used in various fields [24,34,35,36,37]. Besides the definite hydrophobic interior, the structure of cyclodextrins also offers a hydrophilic environment owned by the multiple hydroxyl groups on both rims of cyclodextrins. Depending on the envisioned strategy, primary or secondary hydroxyl groups can function as reactive sites for covalent modification. Presence of the vicinal secondary diols at the lower rim of β–CD suggests the utilization of the boron–based reaction with the formation of dynamic covalent interaction [38]. This specific covalent association between boron acids and hydroxyl groups is reversible and has extensively been applied in the design of synthetic carbohydrate receptors or sensors [39,40]. Despite that in case of cyclodextrins, the lower rim trans–1,2–diols possesses poorer affinity toward boronates in comparison to the cis–1,2–diols or 1,3–diols [41], there are several reports on the effective combination of boronic acid derivatives with cyclodextrins. Willner and co–workers [42] reported the preparation of β–CD–functionalized CdSe/ZnS quantum dots for optical and chiroselective sensing of different substrates. In their design, the β–CD units were successfully linked to the boronic acid ligands from the surface of the quantum dots via the secondary vicinal hydroxyl groups of the sugar units. In another report, Liu et al. [38] performed a complex study on the mechanisms for the interaction of β–CD trans–1,2–diols with a diboronic acid–containing tetraphenylethene moiety. They have confirmed the dynamic nature with the low binding constant and have proved the selective and cooperative binding of two boronic acid moieties of the tetraphenylethene molecule to two pairs of alternative diols on a β–CD.

In the current work, we describe facile synthesis, characterization, and antimicrobial properties of β–CD functionalized supramolecular hydrogel “reticulated” by the formation guanosine quadruplexes (G4–CD). The presence of seven pairs of 1,2–diols in the structure of β–CD allows for the controlled attachment of up to seven guanosine units by utilizing benzene–1,4–diboronic acid linker, thus offering a tuning tool for the final hydrogel properties studied by rheological measurements. The number of attached guanosine moieties strongly affects the internal structure of the hydrogels observed by the circular dichroism (CD), scanning electron microscopy (SEM), and atomic force microscopy (AFM) studies. Besides, multiple hydrogen bonding throughout the system, tuned by the number of guanosine molecules, offers the thixotropic and injectable properties to the final hydrogel. Additionally, all the prepared hydrogels were assessed for antimicrobial activity against eight reference strains in view of the potential use of the described systems for biomedical applications.

## 2. Results and Discussion

### 2.1. Synthesis of G4–CD Hydrogels

**Scheme 1:**
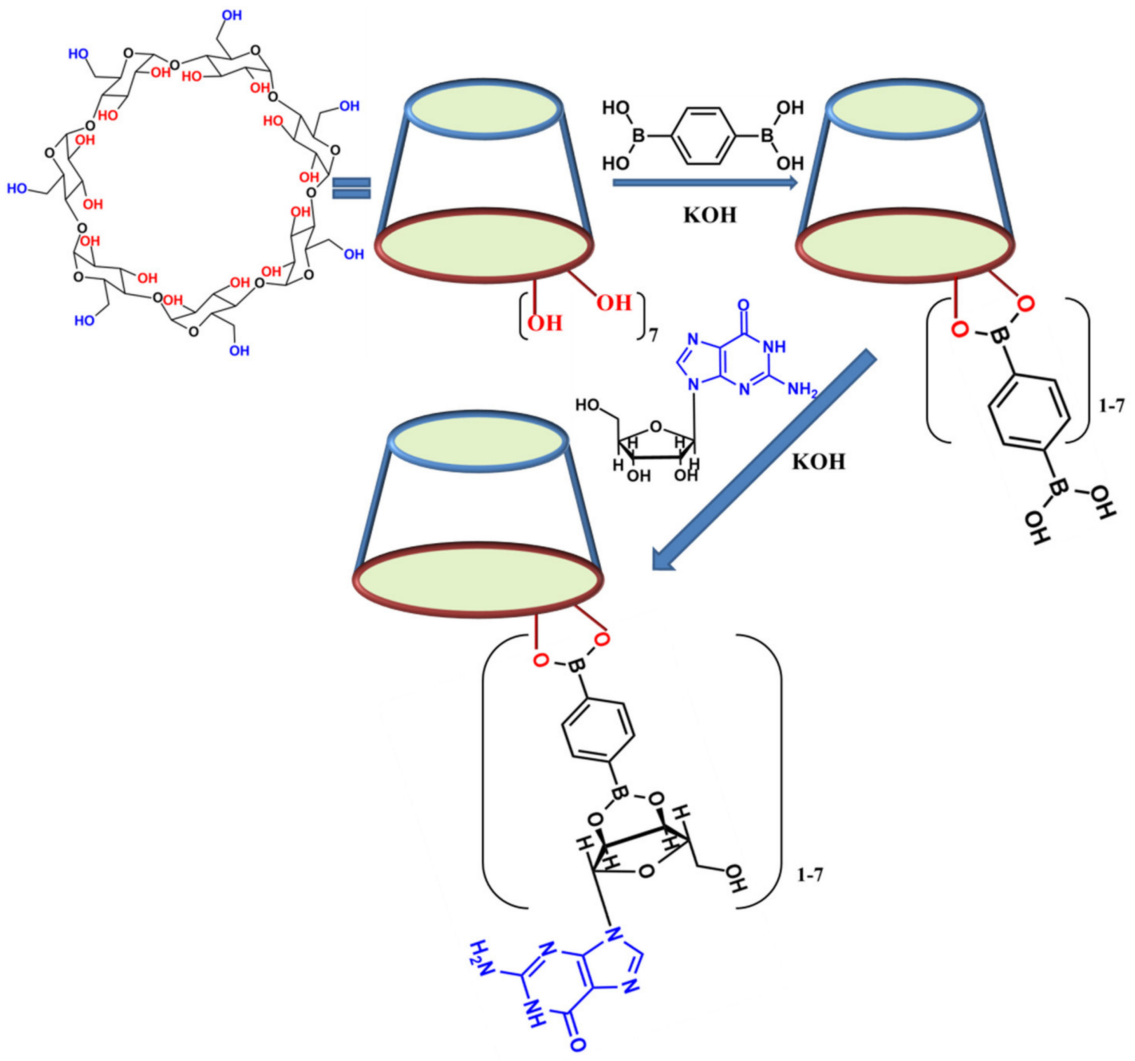
Schematic representation of the G4–CD structural unit synthesis: reaction of the β–CD 1,2 vicinal diols with corresponding equivalents of benzene–1,4–diboronic acid in the presence of KOH, followed by the subsequent interaction of the second boronic acid moiety with the 1,2 diols of the guanosine molecule.

Seven G4–CD hydrogels were synthesized in two–step procedure (Scheme 1), starting by reacting the equivalent amount of benzene–1,4–diboronic acid linker with β–CD in the presence of KOH. The successful reaction between β–CD and boronic acid linker via the secondary vicinal hydroxyl groups of the sugar units under similar conditions was previously reported [38,41,42].

**Figure 1:**
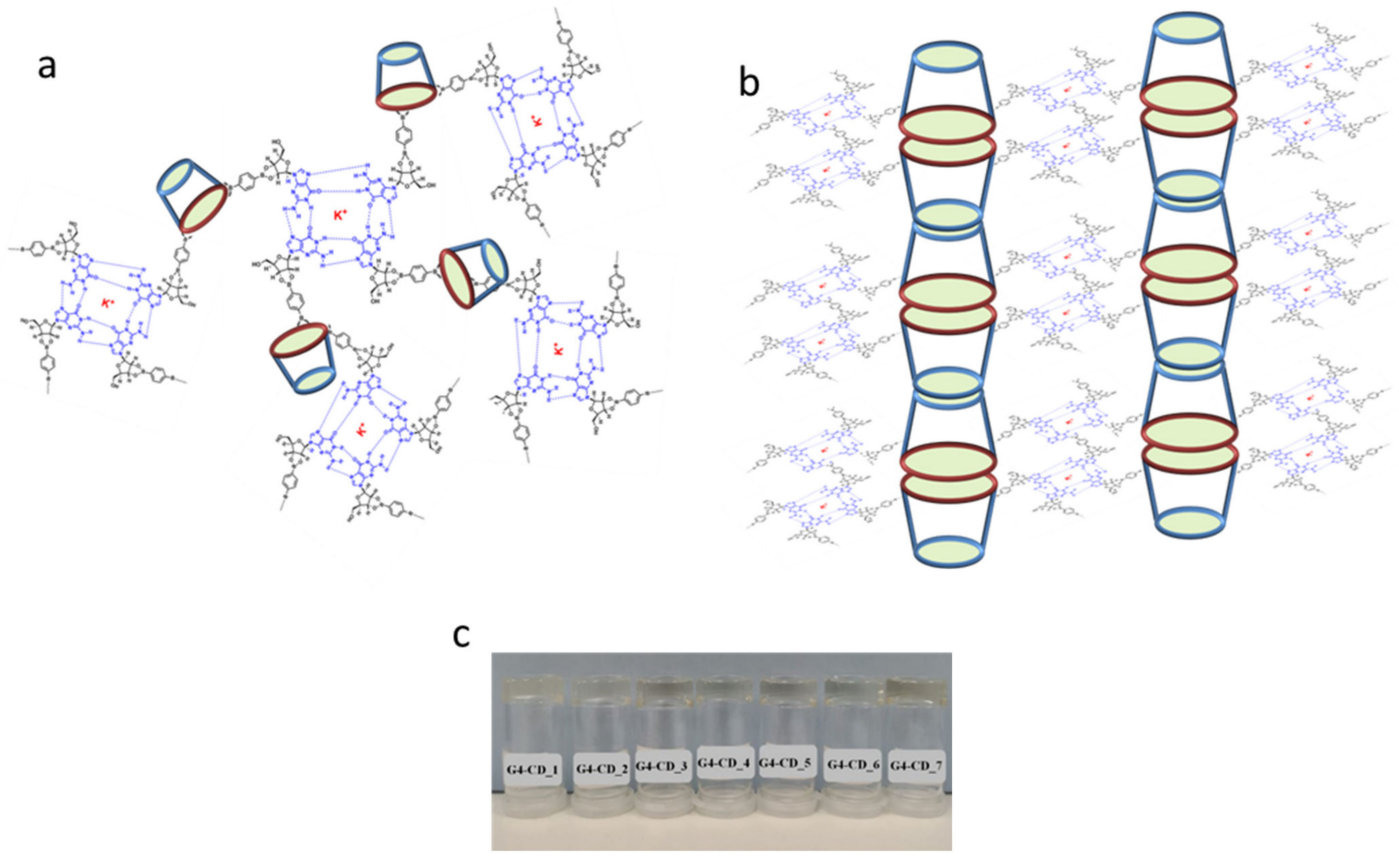
Example of the G4–CD hydrogels network formation (**a**), each guanosine moiety linked to the β–CD molecule through the benzene–1,4–diboronic acid linker participate in the formation of G4 in the presence of potassium ion; possible stacking of β–CD molecules (**b**), driven by the multiple G4 formations; (**c**) picture of self–standing G4–CD_1–7 hydrogels with varied concentration of β–CD.

The boronic ester formation with the 1,2 diols of the β–CD lower rim occurred within minutes at elevated temperature (90 °C) in the presence of an equivalent amount of the base (KOH) to allow the interaction of only one boronic acid moiety with cyclodextrin diols. Subsequently, to the obtained transparent solution, the equivalent amount of guanosine and KOH was added *in situ*, maintaining the same temperature value. At this step, the 1,2 diols of the guanosine sugar moiety reacted with the free boronic acid group of the linker to yield the designed guanosine–functionalized β–CD units according to Scheme 1. Depending on the number of guanosine units, we obtained seven hydrogel systems named G4–CD_1–7. The presence of potassium ions in the reaction mixture led to the arrangement of the guanosine moieties into G4 structures and, correspondingly, to an extended network of linked G4 and β–CDs with the formation of the self–sustained and transparent G4–CD hydrogels (Figure 1).

As previously reported [29,32], the formed G4 tend to stack into guanosine quadruplexes, thus stabilizing the overall hydrogel structure. The number of guanosine moieties in the G4–CD hydrogels strongly influences the number of formed G4 units, thus considerably influencing the mechanical properties of the final hydrogel. In the case of G4–CD_1–3, the hydrogel formation was observed after overnight incubation at room temperature, while samples G4–CD_4–7 presented firm structures after 30 min of incubation at room temperature. We also noticed that G4–CD_1 sample, containing the highest amount of β–CD, turned cloudy within hours due to β–CD crystallization. In contrast, samples G4–CD_2–7 presented stable and transparent hydrogels probably due to the higher substitution of β–CD with the guanosines and thus creation of a more extended network hydrogel internal network. These observations were further confirmed by the AFM and SEM analyses of the samples.

### 2.2. Circular Dichroism Investigations of G4–CD_1–7 Hydrogels

**Figure 2:**
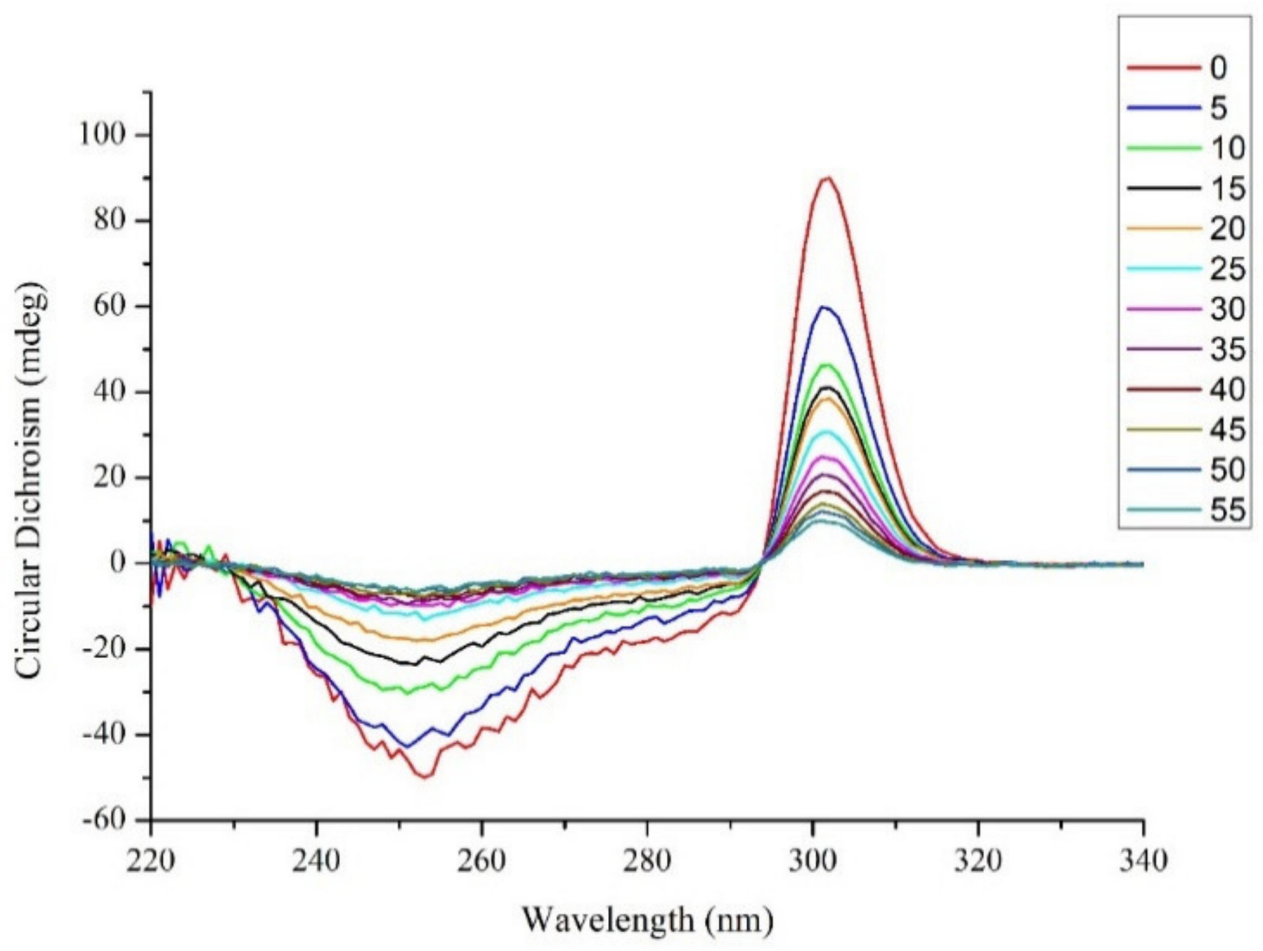
CD spectra of G4–CD_1 hydrogel in the 220–340 nm range recorded every five minutes from 60 to 25 °C.

To gain insight into the formation of the G4–CD_1–7 hydrogel internal structure, we recorded circular dichroism (CD) spectra of each sample taken directly from the reaction mixture in cuvettes with the lightpass of 0.1 mm. Moreover, the CD spectra of warm samples (60 °C) were recorded every five minutes during cooling down to room temperature and stabilization (55 min) to observe the temperature dependent structural changes in the range between 220 and 340 nm (Figure 2 and Figures S1–S7).

Although all the investigated samples displayed individual behavior depending on the amount of guanosine moieties attached to the β–CD molecule, similarities were common for all samples (Figure 1 and Figures S1–S7). Thus, all hydrogels presented strong and sharp positive or negative peaks around 300 nm and a broad negative peak around 250 nm with a shoulder around 270 nm. Samples G4–CD_4–7 presented a small broad positive peak at 225 nm, unlike samples G4–CD_1–3. Additionally, the in–time intensity deviations for the negative band at 251 nm were opposite to that of the positive 303 nm band in the case of G4–CD_7 (Figure S7). A decrease in both positive and negative bands for sample G4–CD_1 (Figure S1), a decrease of negative 251 nm band intensity accompanied by increase of positive 303 nm band for G4–CD_3, simultaneous increase in both negative 251 nm and 303 nm for G4–CD_2, and an unclear dependence of both 251 nm and 303 nm bands in the case of G4–CD_4–6 were observed. Samples G4–CD_2 and 6 first displayed strong positive bands at 303 nm, followed by an impressive change into strong negative peaks upon cooling to room temperature. Typically, the high intensity positive and negative peaks at ~300 nm are characteristic for more complex systems [43] in which heavy G4 aggregates display signals higher than 300 nm. A negative circular dichroism band at 303 nm observed for samples G4–CD_2 and 6 is an unusual signal for G–quadruplex structures, and may be attributed to several different types of associated structures [44].

In the 220–290 nm range, negative broad band was observed around 250 nm in all the investigated samples at room temperature (Figures S1–S7). In the case of sample G4–CD_1 (Figure S1) the band intensity at 251 nm is stronger at higher temperatures with a decrease of intensity in time during cooling to room temperature. Only during the investigation of this sample, the decrease of this band intensity is also accompanied by the intensity decrease of the 303 band. Conversely, samples G4–CD_2, 5 and 6 displayed one broad positive band at higher temperatures (Figures S2, S5, and S6), which gradually transformed into a negative band around 250 nm with the increase in intensity during cooling to room temperature. The intensity of samples G4–CD_3, 4 and 7 showed only the negative broad band around 250 nm of different intensities (Figures S3, S4 and S7).

The CD bands maxima observed for all the investigated samples are in disagreement with the previously reported data for the telomeric sequences and other G–rich species [44,45], exhibiting characteristic negative band at 260 nm and a positive band at ~290 nm. The behavior of the G4–CD_1–7 systems is closer to the assembly of guanosine analogues [46], which self–assemble into G4–based structures, particularly the guanosine 5’–monophosphate derivatives [44] in which a mixture of assemblies, structural motifs, or conformers existing simultaneously in a solution was observed and investigated. These types of guanosine derivatives exhibit CD bands with maxima depending strongly on the sample concentration, temperature, and presence of multiple species in solution. Conversely, presence of strong positive or negative bands around 300 nm in G4–CD samples was not observed in similarly assembled guanosine–based hydrogels [29,32], suggesting strong involvement of the β–CD molecule in the assembly mechanism of the G4–CD_1–7. We speculate the stacking of the β–CDs driven by the guanosine moieties (Figure 1), leading to the possible formation of the cyclodextrin–guanosine wires or fibrils. The possible formation of the hybrid wires will be further discussed by the AFM results.

### 2.3. Scanning Electron Microscopy

**Figure 3:**
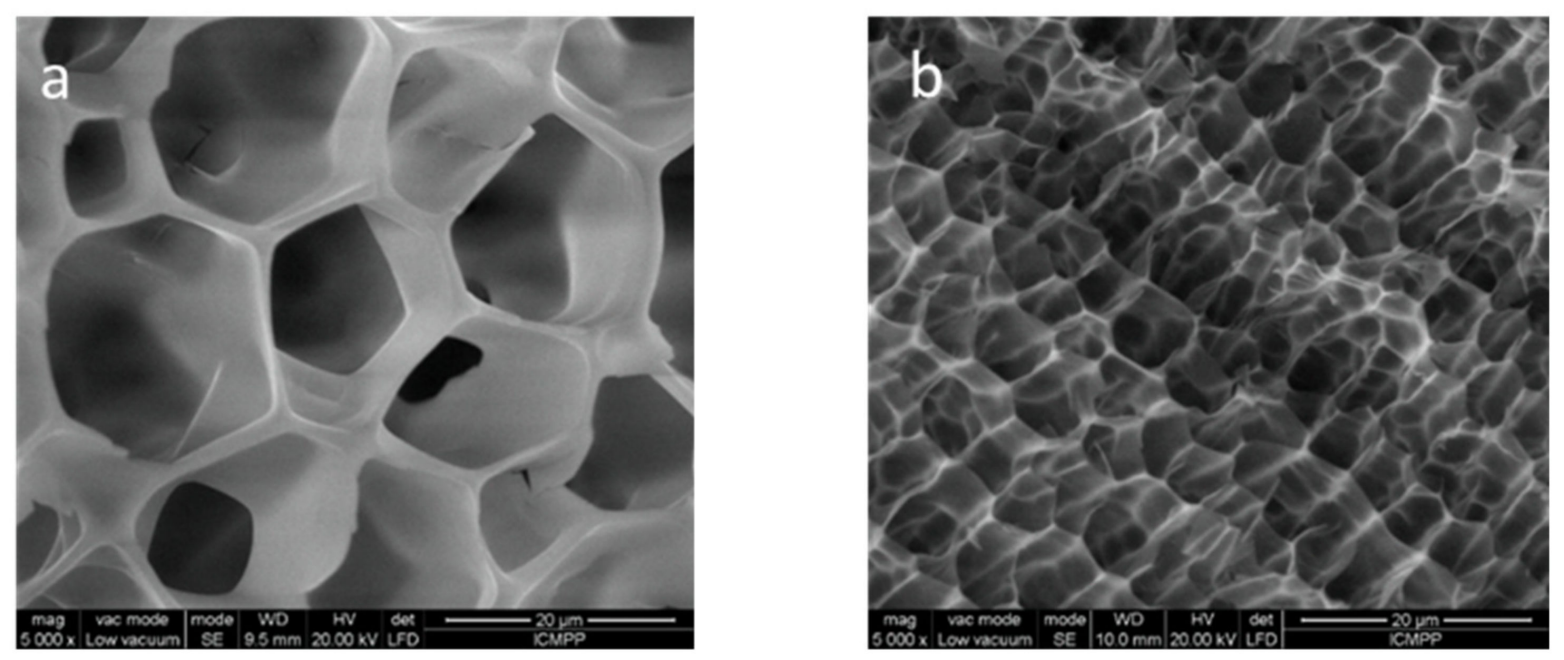
Representative SEM images of freeze–dried G4–CD_1 (**a**) and G4–CD_7 (**b**) hydrogels reflecting differences in pore size and wall thickness.

The morphology of G4–CD_1–7 hydrogels was studied by SEM on lyophilized hydrogel samples (Figure 3 and Figures S8–S14). The investigated SEM images show that the cross–section morphology and porosity of the hydrogel network were strongly influenced by the content of the β–CD and guanosine in the sample. All samples possessed uniform porous structures with differences in the size and thickness of the pores’ walls. G4–CD_1 sample (Figure 3a and Figure S8) had the largest pore size and the thickest pore walls, which are consistent with its lowest amount of guanosine suitable for network crosslinking. Contrarily, sample G4–CD_7 (Figure 3b and Figure S14) displayed the smallest pore size and the thinnest pore walls probably due to the highest degree of the β–CD substitution, with guanosine able to participate G4 quadruplex formation, thus extending the hydrogel network.

The decrease in pore size with the increase in guanosine content of the sample can be observed in all samples except G4–CD_2 (Figure S9). This sample shows pores size similar to samples G4–CD_6, 7 rather than to G4–CD_1, 3. The sample preparation and SEM analysis for G4–CD_2 was repeated but with the same result. Unfortunately, due to the complexity of the system, we have no reasonable explanation to the observed network formation.

### 2.4. X–ray Diffraction

**Figure 4:**
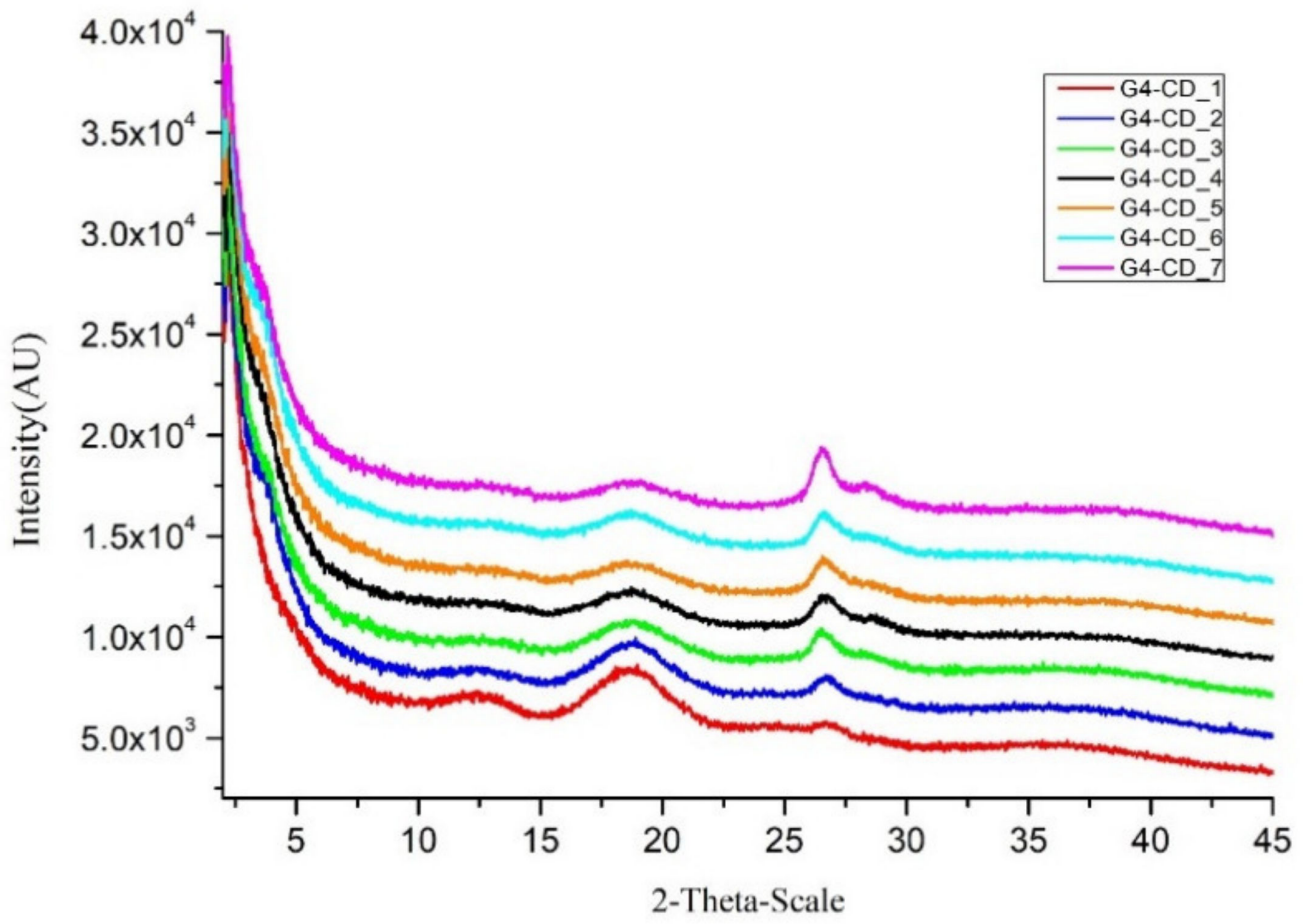
Powder X–ray diffraction patterns of freeze–dried G4–CD_1–7.

To further investigate the assembly mechanism of the G4–CD_1–7, we performed the structural analysis by X–ray diffraction (XRD). Hydrogels G4–CD_1–7 were lyophilized to form white powders, and X–ray diffraction analysis was performed using CuKα–emission in the angular range 2–50° (2θ) with a scanning step of 0.01° and a recording rate of 2°/min. The data obtained are represented in Figure 4.

The XRD data for all the investigated lyophilized samples showed the presence of two main reflections (18.7° and 26.6°) that correspond to the intermolecular d–spacing 3.3 and 4.7 Å, respectively (Figure 4). The presence of the halo XRD pattern with a broad peak at 18.7^o^ and the absence of the characteristic crystalline β–CD diffraction peaks [47] indicated the amorphous structure of G4–CD_1–7 samples which, indicated the channel–type assembly of the β–CD molecules within the sample [48,49]. Typically, in channel–type assemblies, the β–CD molecules are aligned and stacked in a head–to–head arrangement, forming long cylindrical channels through intermolecular hydrogen bonding interactions between the hydroxyl groups of the β–CD molecules [49,50].

The peak at 26.6° represents a characteristic peak describing the π–π distance between G–quartet stacks in previously reported related systems [29,32]. The peaks intensity of signals at 18.7 and 26.6° strongly depend on the ratio between β–CD and guanosine in the structure of the sample (Figure 4). Thus, the decrease of the β–CD amount in the sample is accompanied by the decrease of the 18.7° intensity and, correspondingly, the increase in the 26.6° signal due to the multiple formation of the G4 structures.

The XRD data strongly support the circular dichroism assumptions on the formation of large aggregates based on the G4 structure and aligned β–CD arrangements. To further confirm the formation of long assemblies, we performed detailed AFM investigations of the G4–CD_1–7 samples.

### 2.5. Atomic Force Microscopy

**Figure 5:**
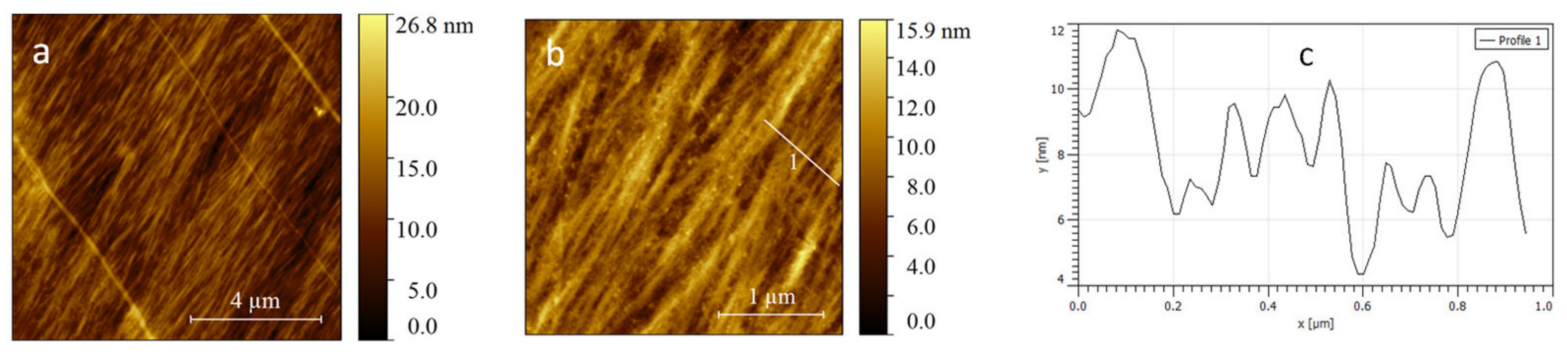
AFM images of G4–CD_3: (**a**) scale bar–4 µm; (**b**) scale bar–1 µm; and (**c**) Z–profiles along the lines marked on the image (**b**).

Insights on morphological structures of hydrogels G4–CD_1–7 were acquired by AFM. All investigated samples presented inter–connected fibrils of several micrometers in length, with different fibril thickness (Figure 5 and Figures S15–S19).

Since this type of structuring was not observed on previously reported G4 hydrogel systems [29], we assumed strong involvement of β–CD molecules over the formation of fibrils. Thus, for hydrogels with the highest amounts of β–CD (G4–CD_1–2), only the thin fibrils were present. Together, with large aggregates and square crystal shapes specific to unreacted CDs, could be observed (Figure S15). The appearance of the clearly formed fibrils was more evidenced, starting with G4–CD_3 (Figure 5) in which the height profiles could be easily measured. Moreover, beginning with G4–CD_3, the height map images analysis illustrated the presence of spherically shaped nanoparticles with mean diameter of about 30 nm that were partially dispersed along the hydrogel fibrils. As the amount of the guanosine substituents on the β–CD increased (G4–CD_4–7), a variation of fibrils diameter (from 90 to ~110 nm) and the growth length of the fibrils, together with overall organization of the hydrogel internal network, were evidenced.

### 2.6. Rheology Investigations

**Figure 6:**
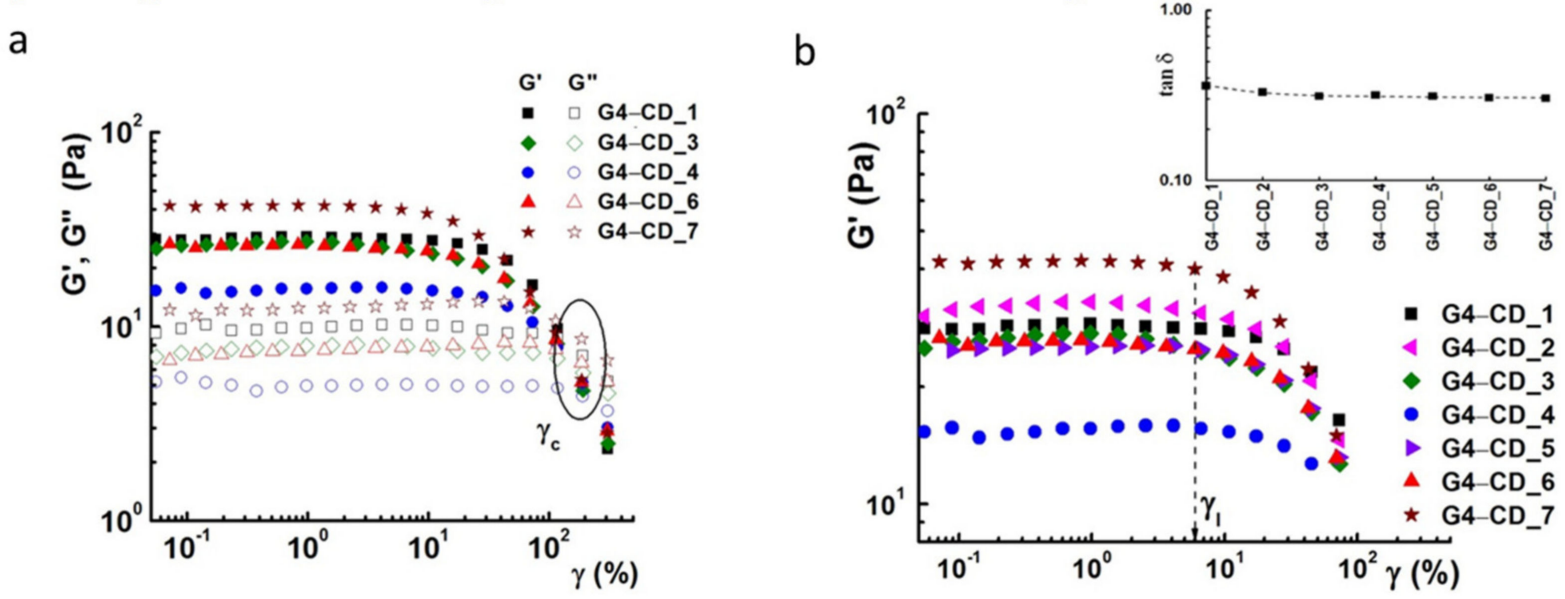
Variation of (**a**) the viscoelastic moduli and (**b**) G’ as a function of strain, γ, at 25 °C and 10 rad s^−1^. The inset figure illustrates the evolution of tan δ at 1 Pa and 10 rad s^−1^ for all samples.

All the investigated G4–CD_1–7 samples revealed gel–like behavior, characterized by storage modulus (G’) higher than loss modulus (G”) (Figure 6a).

The samples were stable up to a critical strain (γ_c_), located in the range of 150–250%, when their structure was broken, and they acquired liquid–like properties with G” > G’. The structures started to change after a limit strain (γ_l_), which did not significantly depend on the β–CD content (Figure 6b). The γ_l_ values for all samples were around 6%. The shear stress (τ) values, corresponding to γ_l_ and γ_c_, were around 2 Pa (τ_l_) and 16 Pa (τ_c_), respectively. The interactions between the components of the investigated systems (which gave the gel strength) were weak, and at a rather low value of τ_l_, the network structure started to break.

The differences between the viscoelastic moduli of the investigated samples were not significant (Table 1). Thereby, G’ and G” varied in the range of 15.6–41.6 Pa and 5.02–12.6 Pa, respectively. The viscoelasticity degree of the samples can be discussed on the basis of the loss tangent (tan δ), defined as G”/G’ (the inset in Figure 6b). A slight decrease of tan δ was observed for samples G4–CD_1 to G4–CD_4 after which this parameter remained almost constant regardless of β–CD amount (Table 1).

**Figure 7:**
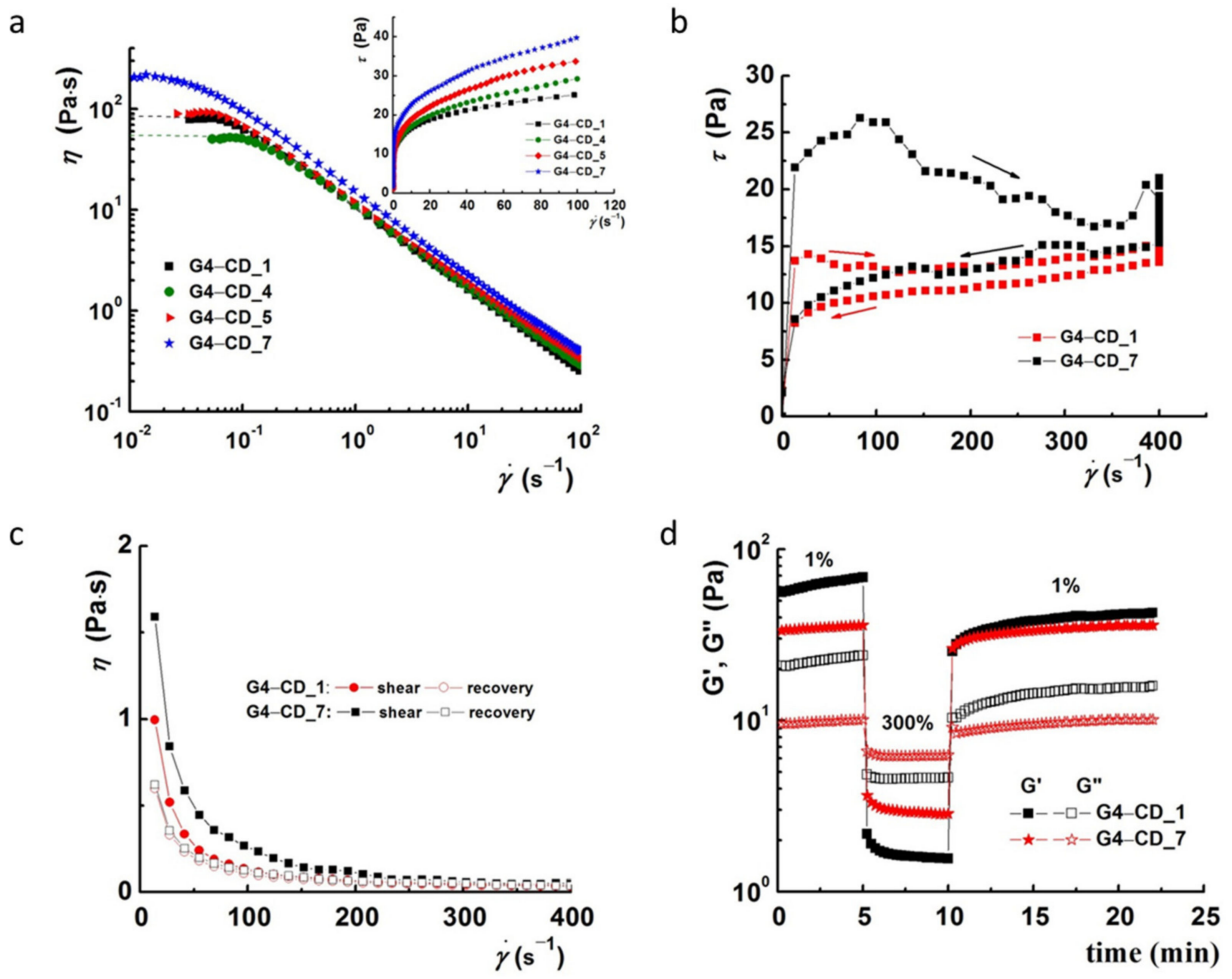
(**a**) Apparent viscosity, η, versus shear rate, $\dot{\gamma }$. The inset represents the variation of τ as a function of $\dot{\gamma }\;$ for investigated samples; (**b**) hysteresis loops for G4–CD_1 and G4–CD_7 samples; (**c**) viscosity recovery at an increase of up to 400 s^−1^ and a decrease to 0 from $\dot{\gamma }$; (**d**) structure recovery ability determined by the continuous step strain measurements (1%–300%–1%) of G4–CD_1 and G4–CD_7 samples at ω = 10 rad s^−1^.

Additionally, all samples exhibited pseudoplastic behavior characterized by the decrease of apparent viscosity (η) with the increase of shear rate, $\dot{\gamma }$ (shear–thinning behavior). For exemplification, the flow curves for four samples were selected and presented in Figure 7a. The samples showed a decrease of η with three orders of magnitude (from about 10^2^ to 10^–1^ Pa·s) by increasing $\dot{\gamma }$ from 10^–2^ to 10^2^ s^–1^. The values of viscosity at low shear rate (η_0_) were determined by fitting the experimental data (η as a function of $\dot{\gamma }$) with the three parameters Carreau model [51] (Table 1). The flow curves, τ versus $\dot{\gamma }$, were non–linear as can be seen in the inset in Figure 7a. The η_0_ values varied between 55.53 and 98.45 Pa·s, except for the sample G4–CD_7, which exhibited the highest value of 217.36 Pa·s.

**Figure 8:**
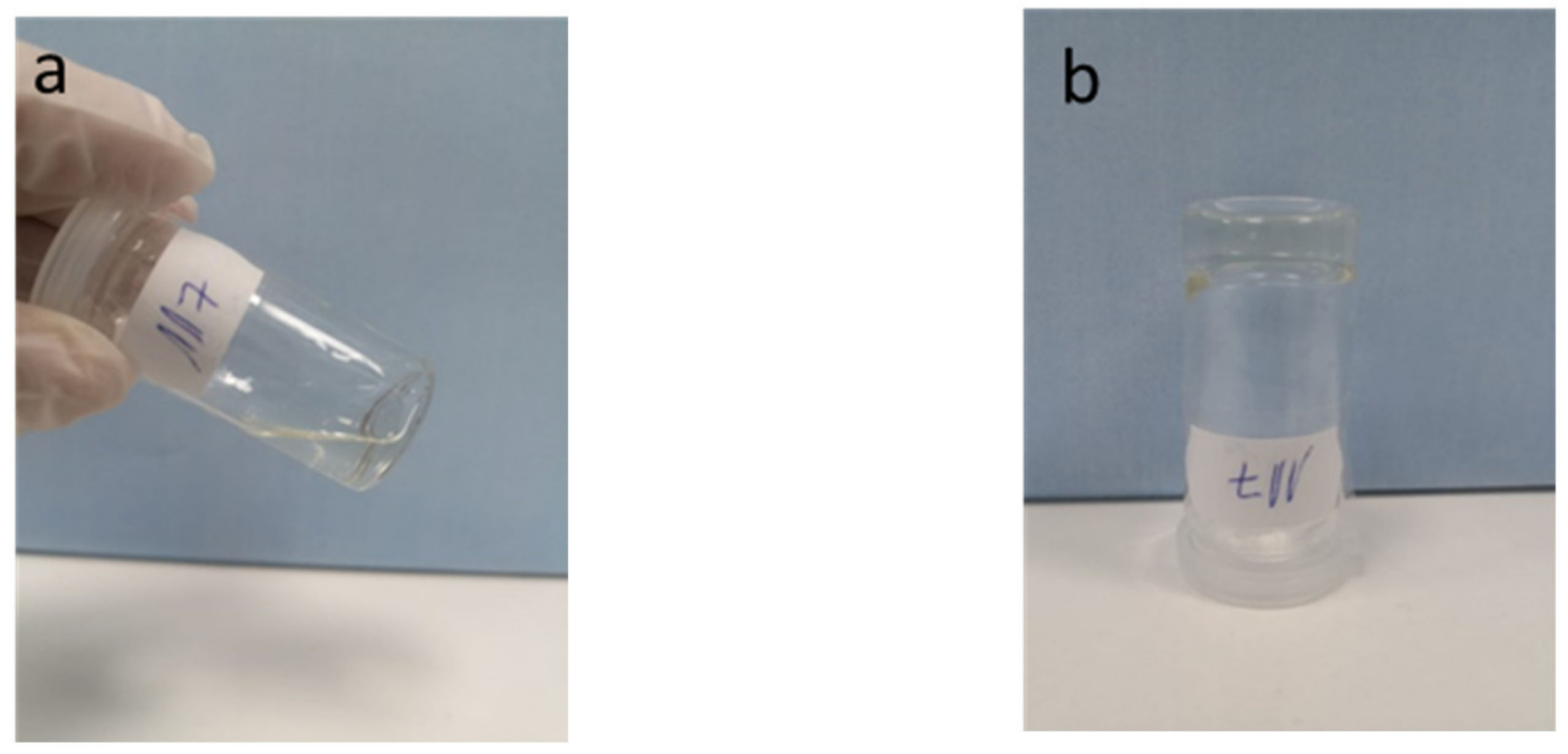
Inversion tube test for G4–CD_7: immediately after mechanical shacking (**a**) and after 100 s left at room temperature (**b**).

All studied samples exhibited thixotropic behavior, characterized by the upward curve above the downward curve in the hysteresis loop. In Figure 7b, the hysteresis loops for two samples (with the lowest and highest β–CD content) are exemplified. The curves present a stress overshoot that can be explained by the requirement to apply a higher stress for starting the flow of the samples. G4–CD_7 shows a higher area of hysteresis loop, indicating that higher stress is necessary to break its structure. This behavior is due to the stronger interactions established between the components of G4–CD_7 sample. We observed that the mechanically shacked hydrogel sample presented a liquid behavior (Figure 8a) but re–establish its firm properties after 100 s (Figure 8b).

The investigated samples also exhibited injectable properties. Figure 7c exemplifies the variation of η by increasing $\dot{\gamma }$ up to 400 s^−1^ in which the sample was sheared for 60 s, and its return for samples G4–CD_1 and G4–CD_7. By applying the shear, the apparent viscosity decreased continuously due to the orientation of assemblies in the flow direction at low $\dot{\gamma }$ and to the destroying of the interactions between them as the shear increased. Viscosities did not fully recover by decreasing $\dot{\gamma }$ because destroyed interactions required a longer time for its restoration (Table 1). The percentage of viscosity regeneration for the investigated samples was found to be between 41.08% and 71.17%.

The structural recovery degree and time represented important requirements for certain biomedical applications (for example, injectable hydrogels). In order to investigate the structural recovery degree, the samples were subjected to a rheological test with three shear steps: (1) the samples were submitted for 5 min to a strain of 1% (from LVR); (2) a strain of 300% (outside LVR) was applied for the next 5 min; (3) the structure recovery was followed, to the strain of 1% for 12 min.

Figure 7d exemplifies the variation of G’ and G” for G4–CD_1 and G4–CD_7. The structure recovery degrees, after 5 min from the stress removal, for samples G4–CD_1 and G4–CD_2, were below 60% while the other samples exhibited a recovery of around 90% (except for G4–CD_6, which showed a recovery of 83.02%) (Table 1).

At the end of the test, the structures of samples with high β–CD content were fully recovered, proving that β–CD presence favored the restoration of the interactions between the system components. In addition, these samples showed a slight increase of viscoelastic moduli under reduced shear, probably because of the new interactions formed (Figure 7d). After 12 min from the stress removal, the structures of the G4–CD_1 and G4–CD_2 samples were only partial recovered, showing a restoration of 62.18% and 71.61%, respectively. For low β–CD content, the samples needed a longer time to re–establish the interactions between the components.

For the G4–CD_7 hydrogel, an experiment to visualize the injectable properties was performed; the results are provided in the video file (V1) in the Supplementary Materials. The tested hydrogel obtained under standard conditions (2 mL) was loaded with fluoresceine (200 µL from stock solution of fluoresceine: 2 mg in 10 mL distilled water) during the preparation to facilitate hydrogel visualization and placed in a 5 mL syringe. After cooling to room temperature, the fluoresceine containing hydrogel was injected into a vial containing the KCl (155 mM) water solution. It could be easily observed that the injected material maintained its inserted shape inside the solution after mechanical shaking.

### 2.7. Antimicrobial Properties

Since the designed G4–CD systems were envisioned for biomedical applications, it was important to assess their antimicrobial properties. The antimicrobial ability of G4–CD_1–7 was evaluated against eight reference strains; the results are summarized in Table S1 and Figure S20.

All the hydrogels did not present antimicrobial activity against most of the investigated reference strains but were efficient against the Gram–positive strain represented by *S. aureus*.

**Figure 9:**
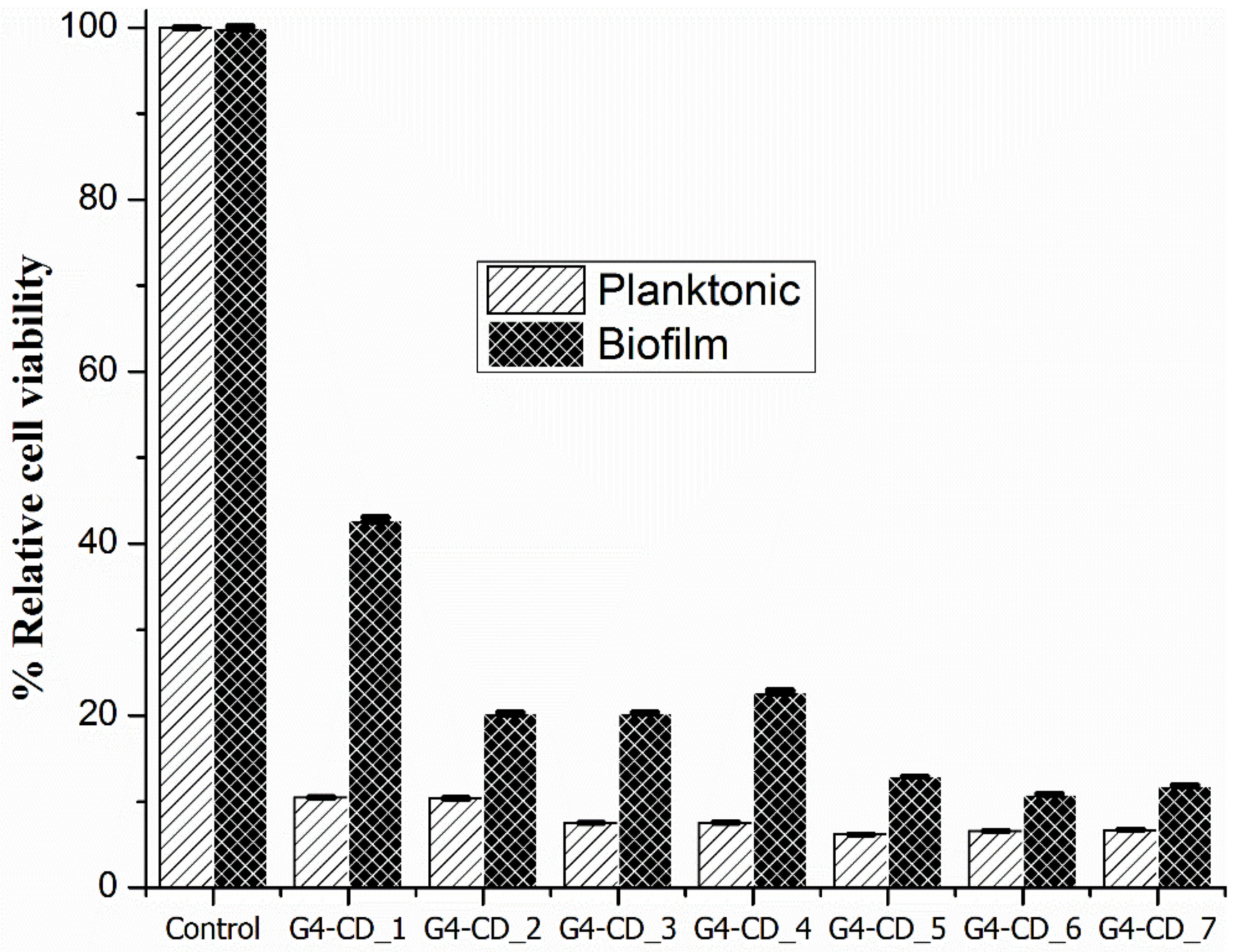
Relative cell viability of *S. aureus* in planktonic and biofilm phase after incubation with G4–CD_1–7 hydrogels.

The observed antibacterial activity of all G4–CD_1–7 hydrogels against *S. aureus* could be explained by the specific action of substituted CDs over the integrity of Gram–positive bacteria membranes observed earlier [52,53]. Particularly, the β–CD molecules participated in K^+^ efflux through definite organizations in bacterial membrane, leading to subsequent complete loss of the K^+^ [52]. This mechanism may also explain the relative cell viability results of *S. aureus* in planktonic and biofilm phase (Figure 9).

According to Figure 9, all the tested hydrogels proved to be efficient against the Gram–positive bacterial strain represented by *S. aureus*, destroying more than 90% of the planktonic cells. The results were different when tested against the bacterial biofilm; only hydrogels G4–CD_5, G4–CD_6, and G4–CD_7 displayed almost the same strong activity similar to the activity against planktonic cells. These data support the assumption that the activity of the G4–CD_1–7 hydrogels against *S. aureus* may be caused by the specific arrangements of the CDs within the hydrogel matrix leading to the afflux of K^+^ through the membrane. The higher the β–CD arrangement in the hydrogel (samples G4–CD_5–7), the higher the observed destroying potential of the hydrogel.

The described hydrogel systems can potentially be loaded with dedicated antibiotics able to form stable inclusion complexes with cyclodextrins [54,55,56]. This strategy may enhance the already observed antibacterial activity of the hydrogel by increasing the stability and the solubility of the utilized antibiotics. Moreover, the entrapment of the antibiotics inside the hydrogel matrix through host–guest complexation may result in its slower release rate, thus prolonging the overall therapeutic efficacy.

## 3. Materials and Methods

### 3.1. Materials

The chemicals utilized in this work such as β–CD, benzene–1,4–diboronic acid, guanosine, fluoresceine, LiOH and KOH were purchased from Sigma (Schnelldorf, Germany). All the purchased reagents were used without further purification. Ultrapure water was used throughout the experiments and stock solutions preparation.

### 3.2. Synthesis of Guanosine–Based β–CD Hydrogels (G4–CD)

Synthesis of G4–CD hydrogels was performed by adapting the previously reported synthesis of guanosine–based hydrogels [29,30]. Depending on the amount of the β–CD, we prepared seven hydrogels (G4–CD_1 to G4–CD_7) containing the corresponding ratio between β–CD and guanosine from 1:1 to 1:7. Thus, a stock solution of β–CD in distilled water (565 mg in 5 mL water) was prepared for the addition of the necessary equivalents of β–CD to the composition of the hydrogels. For example, to prepare a G4–CD_1 containing 1:1 ratio between β–CD and guanosine, 1.771 mL of β–CD stock solution (200 mg, 0.176 mmol) were mixed with benzene–1,4–diboronic acid (29.2 mg, 0.176 mmol) in a 10 mL vial, placed in ultrasonication bath for 1 min, following the addition of the KOH solution (100 µL containing 9.8 mg, 0.176 mmol) and heating on an oil bath (120 °C) for 1–2 min until the solution became transparent. Next, guanosine (50 mg, 0.176 mmol) was added to the reaction mixture, followed by KOH solution (100 µL containing 9.8 mg, 0.176 mmol) and the resulted mixture in the vial heated on the oil bath (120 °C) for another 2–3 min until the solution became clear. The vial was left to cool down to room temperature to form steady transparent hydrogel with a total volume equal to 2 mL.

For hydrogels G4–CD_2 to G4–CD_7, the total volume of the reaction mixture was maintained at 2 mL and the quantities of all components were constant except for the β–CD. The necessary equivalents of β–CD for the corresponding ratios were adjusted by the volume of β–CD stock solution in the reaction mixture (0.885 mL for G4–CD_2, 0.591 mL for G4–CD_3, 0.442 mL for G4–CD_4, 0.354 mL for G4–CD_5, 0.294 mL for G4–CD_6, and 0.253 mL for G4–CD_7).

### 3.3. Characterizations

#### 3.3.1. Surface Analyses

The morphology of G4–CD_1–7 hydrogels was studied by SEM and AFM techniques. In the case of SEM (FEI NanoSEM 430, FEI Company, Hillsboro, USA, landing E: 10.0 keV), hydrogel samples were freeze dried prior to measurements, carefully placed onto 25 aluminum plates with double–sided adhesive and investigated at an accelerating voltage of 20 kV.

AFM investigation was conducted using Ntegra Spectra Atomic Force Microscope (NT–MDT Spectrum Instruments, Zelenograd, Moscow, Russia) operated in tapping mode under ambient conditions. Silicon cantilever tips (NSG 10, NT–MDT Spectrum Instruments, Zelenograd, Moscow, Russia) with gold reflecting coating, a resonance frequency of 140–390 kHz, a force constant of 3.1–37.6 N m^−1^, and a tip curvature radius of 10 nm were used. Aliquots of 10 µL gel solution were evenly deposited onto freshly cleaved mica substrates and dried in air at room temperature prior to imaging. AFM image analysis was performed with Gwyddion 2.59 software [57].

#### 3.3.2. Circular Dichroism

Circular dichroism (CD) measurements were conducted on a Chirascan plus (Applied Photophysics Ltd., Leatherhead, Surrey, UK). The CD spectra were recorded between 340 and 220 nm with data pitch 0.5 nm at 200 nm·min^−1^ acquisition speed in 1 mm lamellas. Typically, 100 µL of sample from the hydrogel preparation reaction mixture (90 °C) was poured directly onto a lamella and covered with the second glass to form a uniform film with no air bubbles in between. All samples were recorded directly from the reaction mixture at the starting temperature of 60 °C, with measurements performed every 5 min until stabilization (55 min).

#### 3.3.3. X–ray Diffraction

Hydrogels G4–CD_1–7 were lyophilized to form white powders and X–ray diffraction analysis was performed on a Rigaku Miniflex 600 diffractometer (Rigaku, Tokyo, Japan) using CuKα–emission in the angular range 2–50° (2θ) with a scanning step of 0.01° and a recording rate of 2°/min.

#### 3.3.4. Rheological Measurements of the Hydrogels

The rheological properties of G4–CD_1–7 hydrogels were performed by using a MCR302 Anton–Paar rheometer with plane–plane geometry (25 mm diameter). The rheometer is equipped with a Peltier system, which ensures the temperature control, and a solver trap cover to prevent the solvent evaporation that is used. In order to establish the linear viscoelastic regime (LVR), an amplitude sweep test was realized at a constant oscillation frequency (ω) of 10 rad s^−1^, in the strain (γ) range of 0.05–500% (the shear stress between 0.01–30 Pa). The storage modulus (G’), the loss modulus (G”), and the loss tangent (tan δ), defined as G”/G’ at 1 Pa, were established. The flow curves were determined from measurements in continuous shear regime in the range of shear rates ($\dot{\gamma })\;$ from 10^−2^ s^−1^ to 10^2^ s^−1^. In addition, the hysteresis loops were obtained by continuous shear measurements, which involved first increasing the $\dot{\gamma }$ up to 400 s^−1^, and then keeping the samples at this $\dot{\gamma }$ for 60 s and, finally, decreasing it from 400 s^−1^ to 0. The structure recovery was followed by step shear experiments in which the samples were subjected consecutively to three shear steps, 1%–300%–1%, at 10 rad s^−1^. All rheological measurements were conducted at 25 °C.

### 3.4. Antimicrobial Property of the Hydrogels

For the microbiological assay: Tryptic Soy Broth, Nutrient Broth, and bacteriological agar from VWR Chemicals, Sabouraud Dextrose Broth from Merck, and CellTiter 96^®^ AQueous One Solution Cell Proliferation Assay from Promega, were purchased.

The antimicrobial activity of the hydrogels was determined by disk diffusion assay [58] against eight different reference strains: bacterial strains*Staphylococcus aureus* ATCC25923, *Escherichia coli* ATCC25922, *Enterococcus faecalis* ATCC29212, *Klebsiella pneumoniae* ATCC10031, and *Salmonella typhimurium* ATCC14028, yeast strains represented by *Candida albicans* ATCC10231 and *Candida glabrata* ATCC90028, and the fungal strain *Aspergillus brasiliensis* ATCC9642.

All microorganisms were stored at −80 °C in 20–40% glycerol. The bacterial strains were refreshed in tryptic soy broth (TSB) at 37 °C. The yeast strains were refreshed Sabouraud dextrose broth (SDB), and the fungal strain was refreshed on potato dextrose broth (PDB) at 25 °C. Microbial suspensions were prepared with these cultures in sterile solution to obtain turbidity optically comparable to that of 0.5 McFarland standards. Volumes of 0.2 mL from each inoculum were spread on the Petri dishes. The hydrogels were placed on proper agar plates and 100 µL of the samples was added in 5 mm holes cut into agar media.

To evaluate the antimicrobial properties, the growth inhibition was measured under standard conditions after 24 h of incubation at 37 °C for the bacterial and the yeast strains and after 48 h at 26 °C for the fungal strain. All tests were conducted in triplicate to verify the results. After incubation, the diameters of inhibition zones were measured by using Image J version 1.52 t software [59].

The hydrogels bacterial cytotoxicity after incubation with the *S. aureus*, in planktonic (free floating) phase and after biofilm formation, was assessed by MTS assay using the CellTiter 96^®^ AQueous One Solution Cell Proliferation (Promega, Madison, WI, USA) according to the manufacturer instructions.

Briefly, after 24 h of bacterial incubation with the tested hydrogels, MTS reagent was added 1–3 h prior to absorbance readings. After the formazan formation, the final reading was performed at 490 nm on a FLUOstar^®^ Omega microplate reader (BMG LABTECH, Ortenberg, Germany).

All data were expressed as the mean ± standard deviation of the mean. Statistical analysis was performed using XLSTAT Ecology version 2019.4.1 software [60].

## 4. Conclusions

We reported the design and preparation of seven cyclodextrin containing supramolecular hydrogels whose gelation mechanism is based on the formation of guanosine quadruplexes, thus maintaining the cyclodextrin availability for the possible host–guest interactions. The covalent insertion of cyclodextrin into the hydrogel matrix was performed by the reaction of 1,2 diols of the β–CD lower rim with the benzene–1,4–diboronic acid linker and subsequent attachment of guanosine molecule to the linker. The presence of potassium ions in the reaction mixture led to the arrangement of the guanosine moieties into G4 structures and, correspondingly, to the formation of transparent hydrogels. The obtained hydrogels’ properties strongly depend on the number of guanosine units attached to the cyclodextrin molecules, thus offering a property tuning tool for the possible applications. The increase in the amount of guanosines led to the increase in internal structuration observed by AFM, SEM, and XRD analyses. Additionally, the ratio between guanosine and cyclodextrin in the hydrogel structure strongly influenced the rheological properties of the sample, while the supramolecular nature of the samples offered interesting self–healing and thixotropic properties. All the prepared samples displayed selective antimicrobial properties against *S. aureus* in planktonic and biofilm phase, the activity also depending on the guanosine and cyclodextrin ratio within the hydrogel structure.

## Figures and Tables

**Table 1:** Rheological parameters of the investigated samples.

Sample	G’ ^a^(Pa)	G” ^a^(Pa)	Tan δ a (=G”/G’)	η_0_ ^b^	η_reg_ ^c^ (%)	Structure Recovery ^d^ (%)
G4–CD_1	28.3	10.2	0.36	87.58 ± 1.1	60.16	55.68
G4–CD_2	29.8	9.8	0.33	63.27 ± 0.7	69.04	57.52
G4–CD_3	20.8	6.38	0.32	82.15 ± 1.2	60.54	93.42
G4–CD_4	15.6	5.02	0.31	55.53 ± 0.7	71.17	91.42
G4–CD_5	25.4	7.94	0.31	98.45 ± 1.1	44.77	92.22
G4–CD_6	25.3	7.73	0.31	74.06 ± 0.5	60.24	83.02
G4–CD_7	41.6	12.6	0.31	217.36 ± 0.9	41.08	93.46

^a^ established by the amplitude sweep test at 1 Pa and 10 rad⋅s^−1^; ^b^ determined by applying the three parameters Carreau model: $\eta =\frac{\eta_{0}}{{\left[1+{\left(\lambda \cdot \dot{\gamma }\right)}^{2}\right]}^{\frac{\left(1-n\right)}{2}}}$ where *η* and *η*_0_ represent the apparent viscosity and viscosity at zero shear rate; $\dot{\gamma }$ is the shear rate; *λ* and n are the relaxation time and power index, respectively, characteristics of each material; ^c^ the percentage of the regeneration of viscosity calculated from hysteresis loops considering the final viscosity and the initial as reference; ^d^ determined after 5 min from the stress removal.

## Supplementary Materials

The following are available online at https://www.mdpi.com/article/10.3390/ijms22179179/s1.
