# Supplementary material for: Injectable Thixotropic β–Cyclodextrin–Functionalized Hydrogels Based on Guanosine Quartet Assembly
Source: Int J Mol Sci. 2021 Aug 25;22(17):9179. doi: 10.3390/ijms22179179 (PMC8431444; doi:10.3390/ijms22179179)
Supplement: Supplementary file 1 [file ijms-22-09179-s001.zip › ESI_Rotaru_R1.pdf]

Injectable thixotropic β-cyclodextrin-functionalized hydrogels based on guanosine quartet assembly

Monica-Cornelia Sardaru[1], Irina Rosca[1], Simona Morariu[2], Elena-Laura Ursu[1], Razvan Ghiarasim[1] and Alexandru Rotaru[1]*

[1] *"Petru Poni" Institute of Macromolecular Chemistry, Romanian Academy, Centre of Advanced Research in Bionanoconjugates and Biopolymers, Grigore Ghica Voda Alley 41 A, 700487 Iasi, Romania.*

[2] *"Petru Poni" Institute of Macromolecular Chemistry, Romanian Academy, Natural Polymers, Bioactive and Biocompatible Materials, Grigore Ghica Voda Alley 41 A, 700487 Iasi, Romania.*

* Correspondence: rotaru.alexandru@icmpp.ro

Content

1. CD spectra – **Figure S1-S7**
2. SEM images – **Figures S8-S14**
3. AFM images – **Figures S15 –S19**
4. Antimicrobial activity – **Figure S20, Table S1**

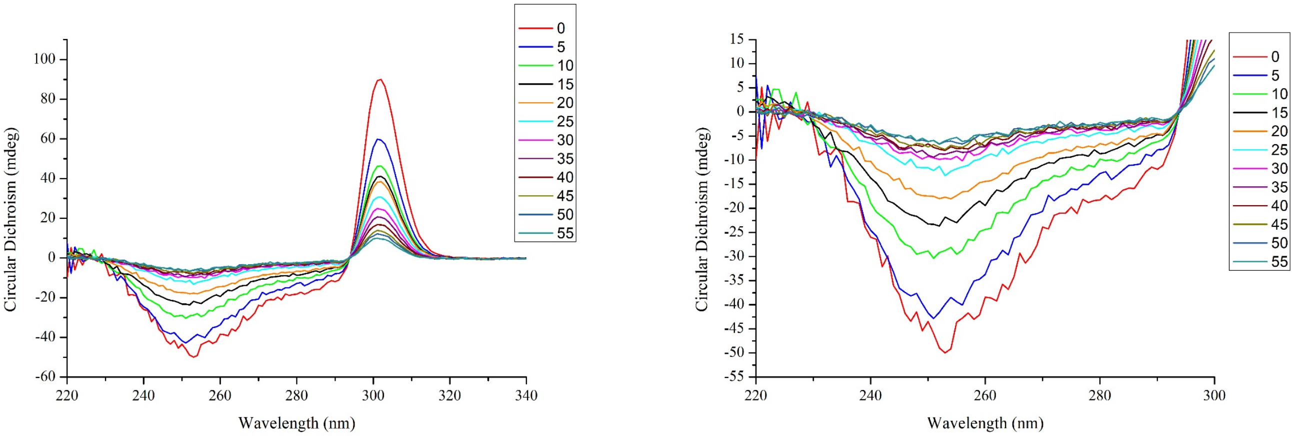

Figure S1. CD spectra of G4-CD_1 in the 220-340 nm range (left) and 220-300 nm range (right) recorded every five minutes from 60 ºC to 25 ºC.

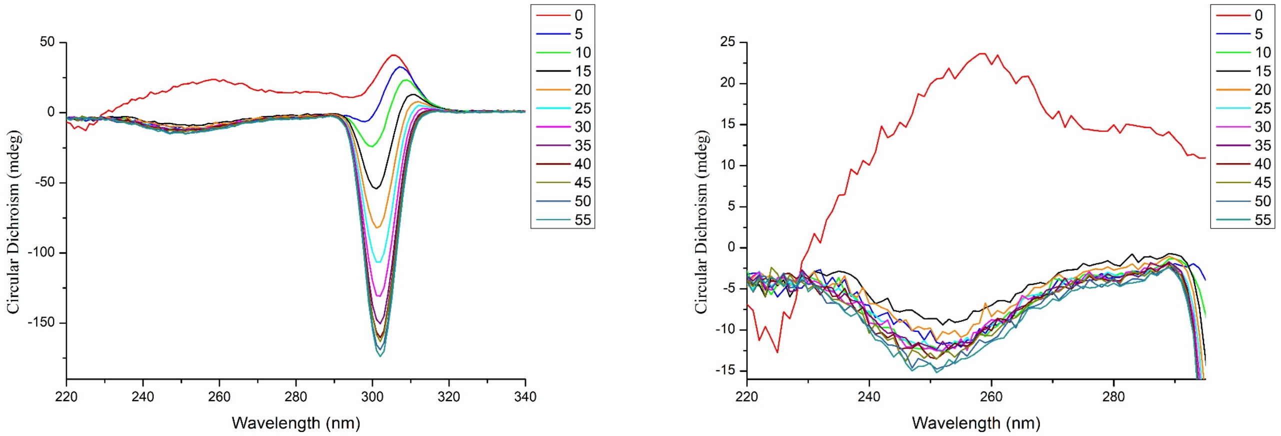

Figure S2. CD spectra of G4-CD_2 in the 220-340 nm range (left) and 220-300 nm range (right) recorded every five minutes from 60 ºC to 25 ºC.

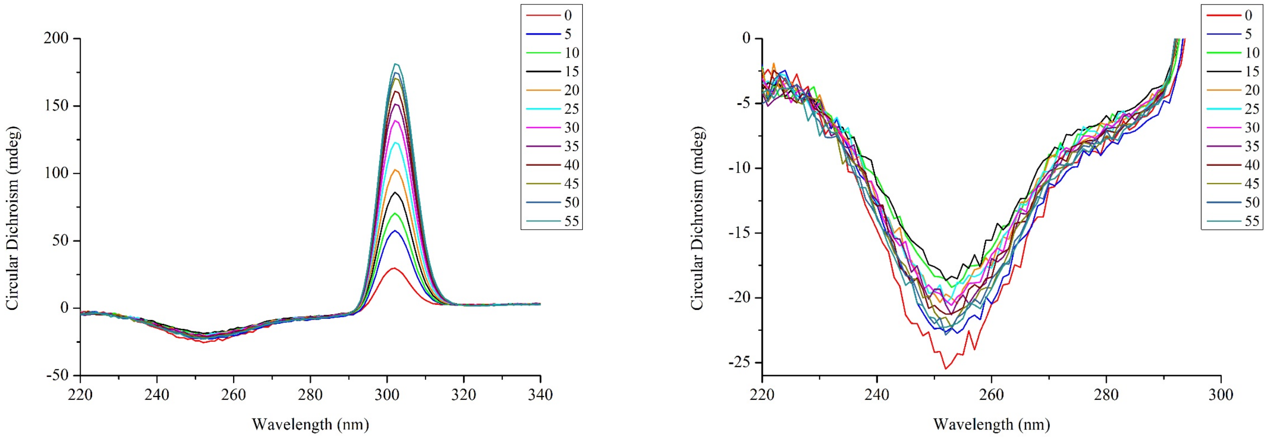

Figure S3. CD spectra of G4-CD_3 in the 220-340 nm range (left) and 220-300 nm range (right) recorded every five minutes from 60 ºC to 25 ºC.

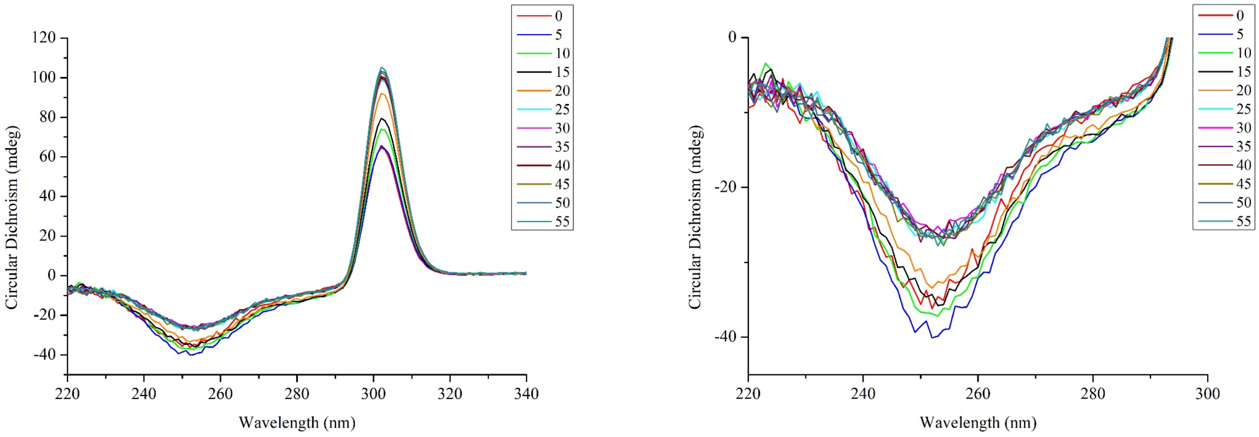

Figure S4. CD spectra of G4-CD_4 in the 220-340 nm range (left) and 220-300 nm range (right) recorded every five minutes from 60 ºC to 25 ºC.

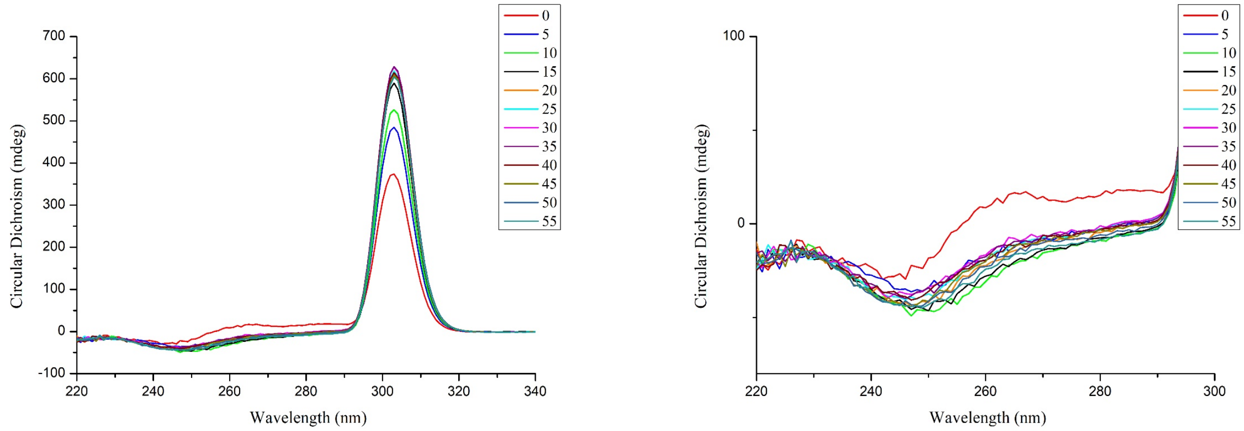

Figure S5. CD spectra of G4-CD_5 in the 220-340 nm range (left) and 220-300 nm range (right) recorded every five minutes from 60 $^{\circ}$C to 25 $^{\circ}$C.

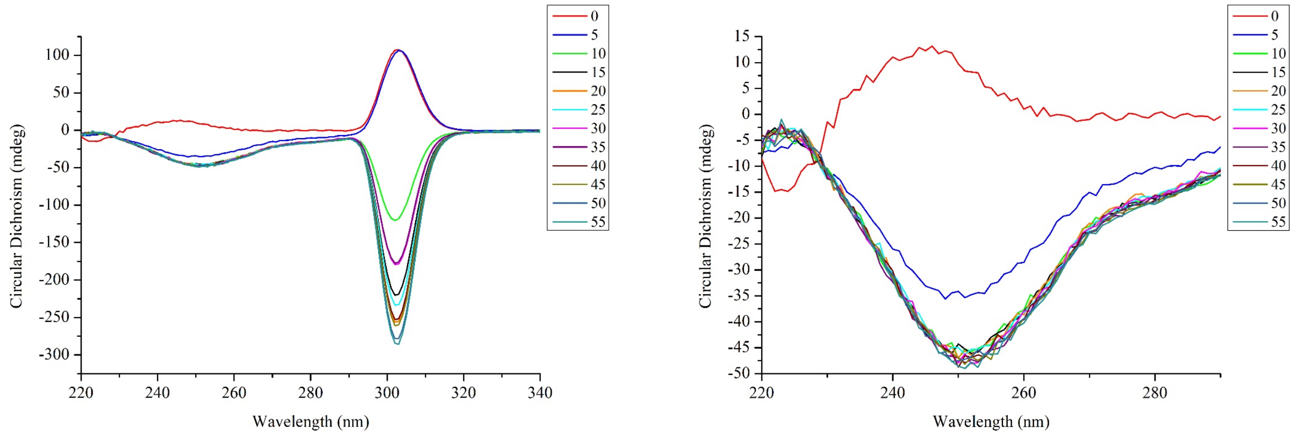

Figure S6. CD spectra of G4-CD_6 in the 220-340 nm range (left) and 220-300 nm range (right) recorded every five minutes from 60 $^{\circ}$C to 25 $^{\circ}$C.

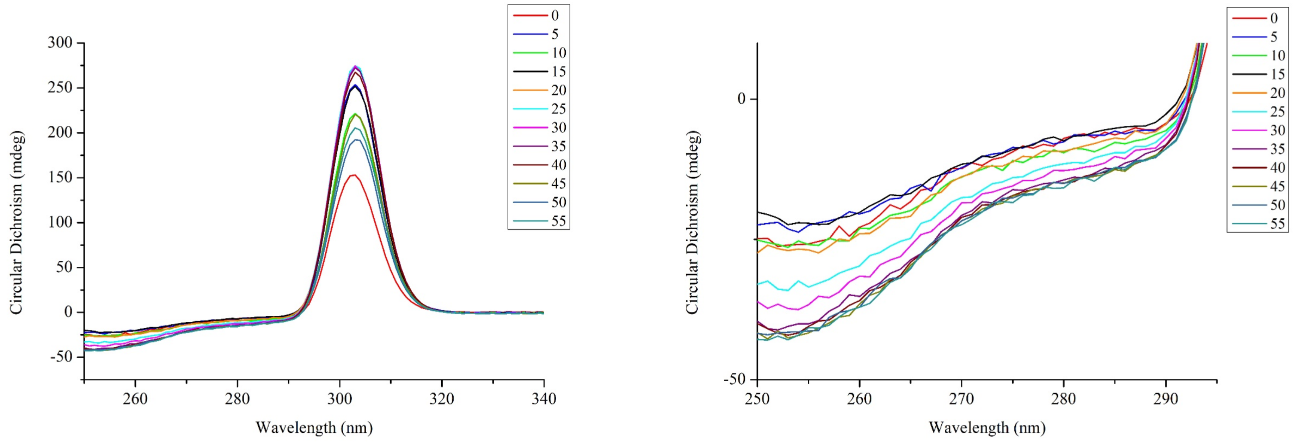

Figure S7. CD spectra of G4-CD_7 in the 220-340 nm range (left) and 220-300 nm range (right) recorded every five minutes from 60 ºC to 25 ºC.

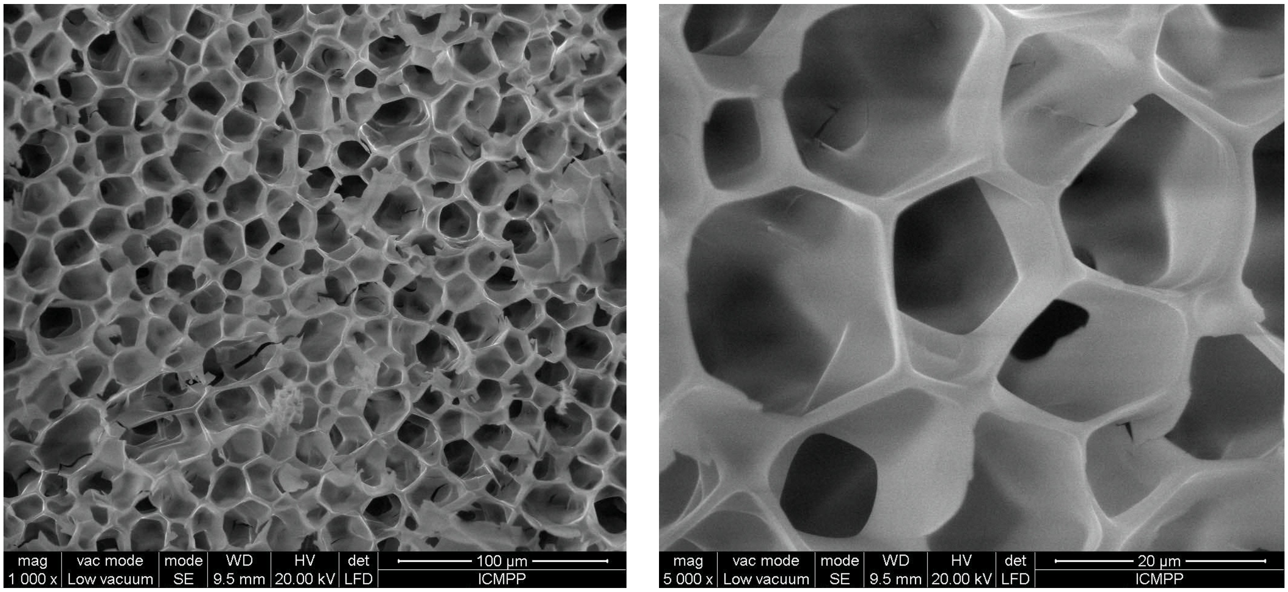

Figure S8. SEM images of G4-CD_1 at different magnifications. Scale bar – 100 µm (left) and 20 µm (right).

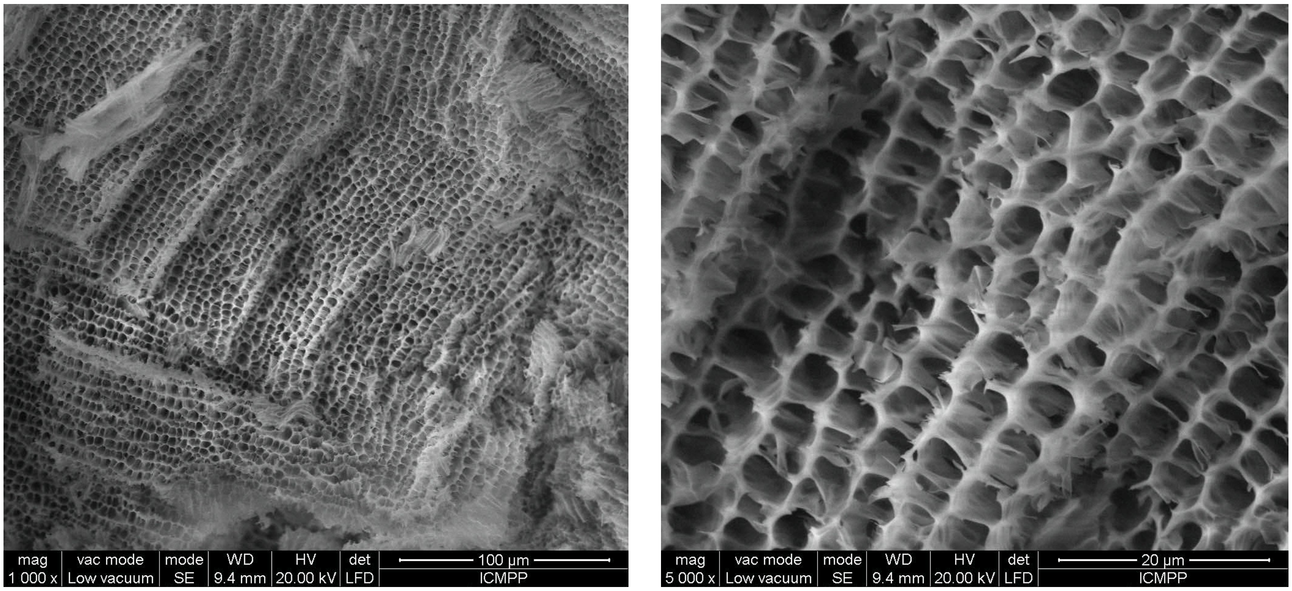

Figure S9. SEM images of G4-CD_2 at different magnifications. Scale bar – 100 µm (left) and 20 µm (right).

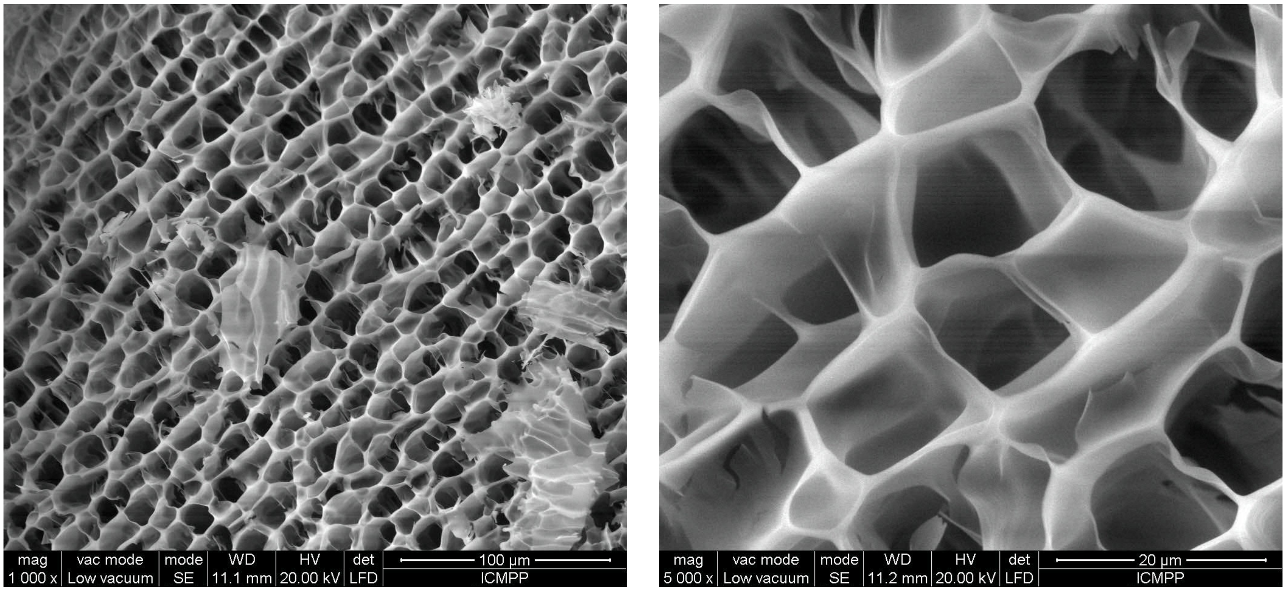

Figure S10. SEM images of G4-CD_3 at different magnifications. Scale bar – 100 µm (left) and 20 µm (right).

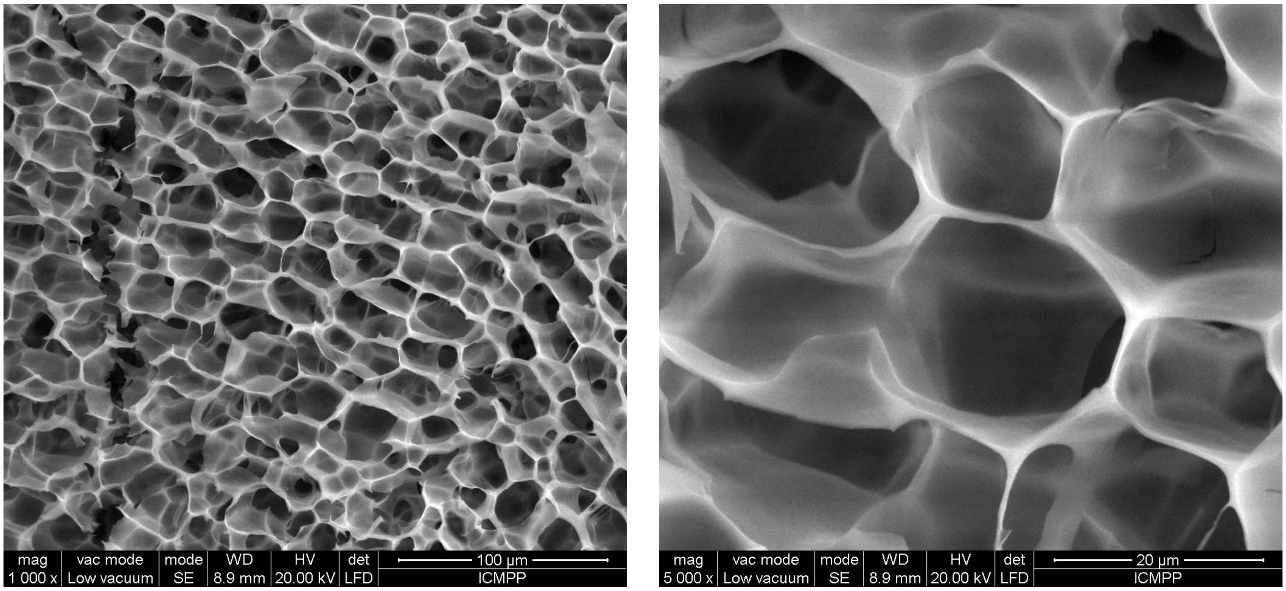

Figure S11. SEM images of G4-CD_4 at different magnifications. Scale bar – 100 μm (left) and 20 μm (right).

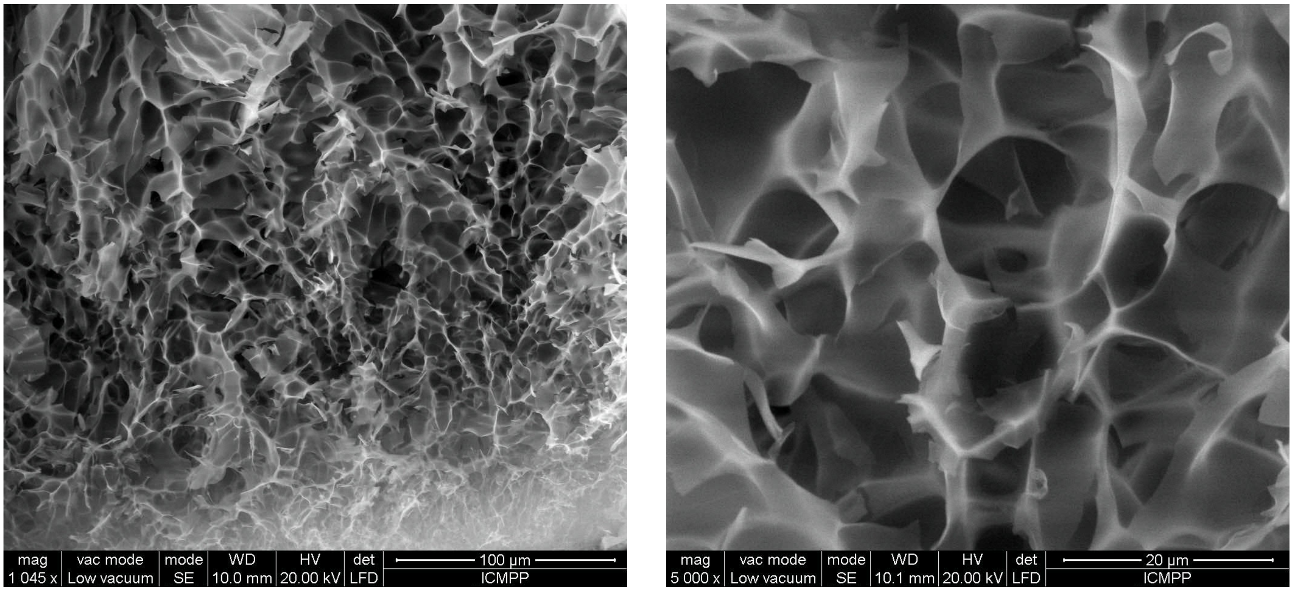

Figure S12. SEM images of G4-CD_5 at different magnifications. Scale bar – 100 μm (left) and 20 μm (right).

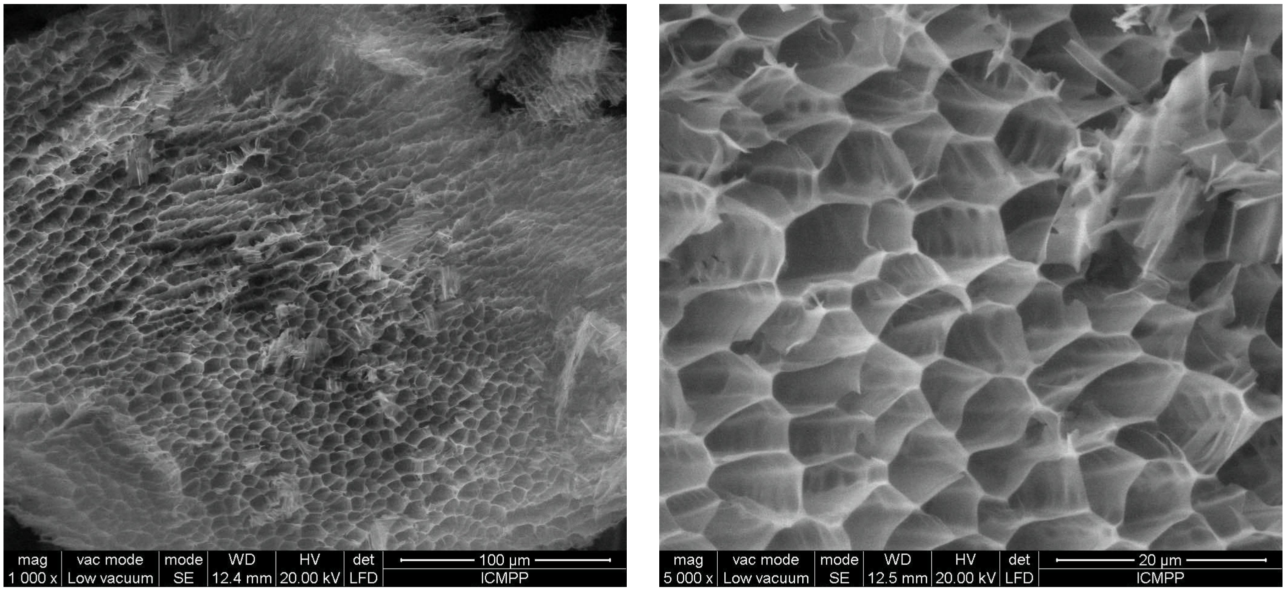

Figure S13. SEM images of G4-CD_6 at different magnifications. Scale bar – 100 µm (left) and 20 µm (right).

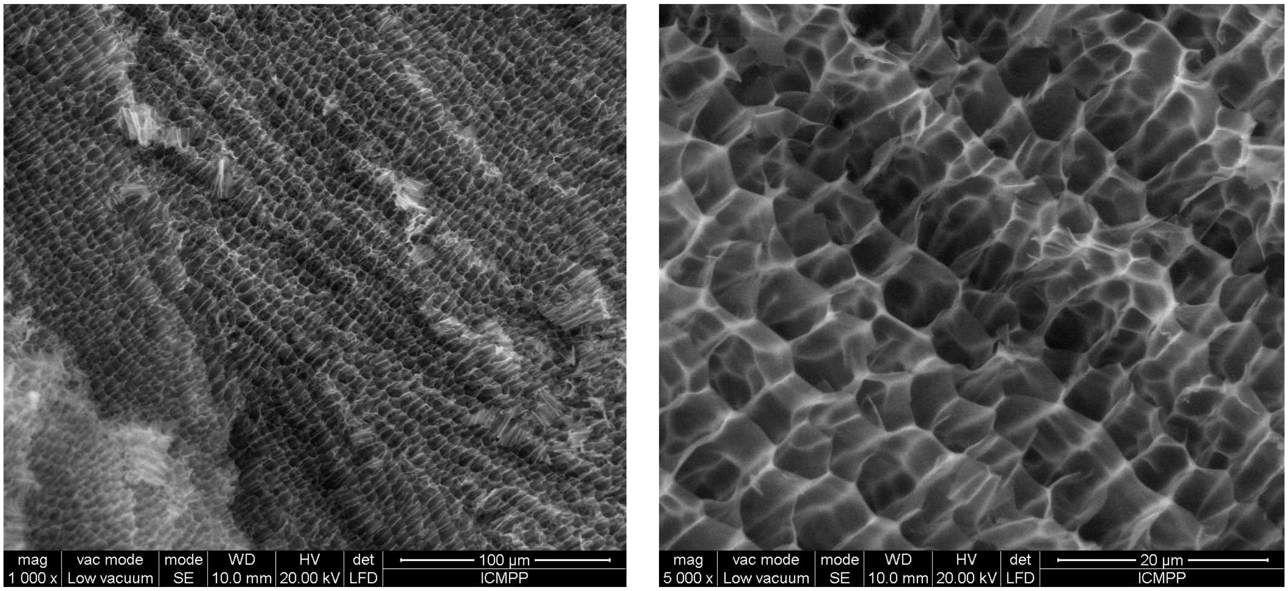

Figure S14. SEM images of G4-CD_7 at different magnifications. Scale bar – 100 µm (left) and 20 µm (right).

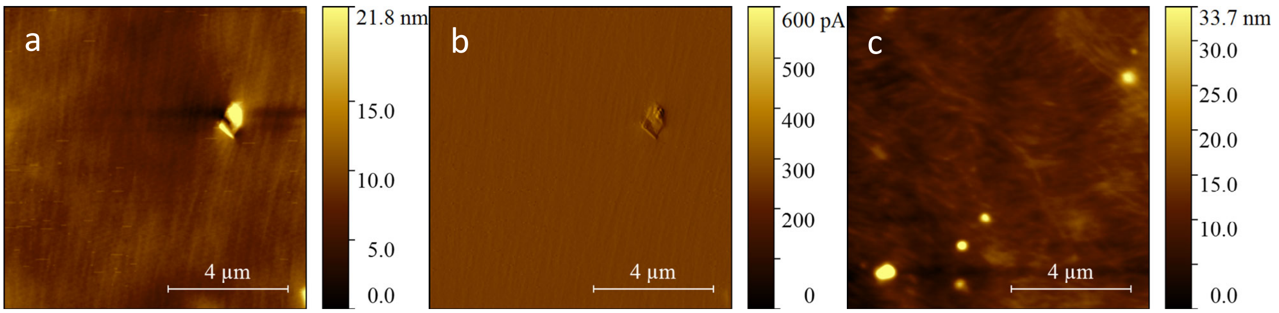

Figure S15. AFM (a) and error signal images (b) of G4-CD_1; (c) AFM images of G4-CD_2: scale bar – 4 µm.

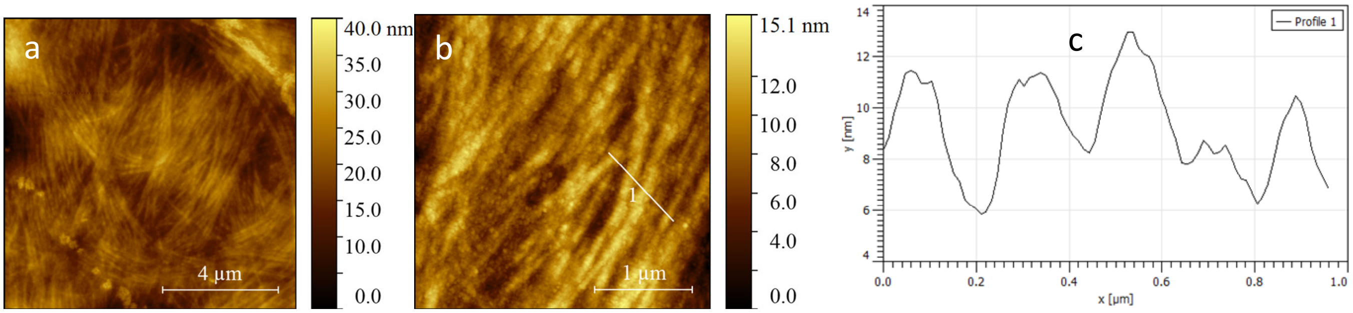

Figure S16. AFM images of G4-CD_4: (a) scale bar – 4 µm; (b) scale bar – 1 µm; (c) Z-profiles along the lines marked on the images.

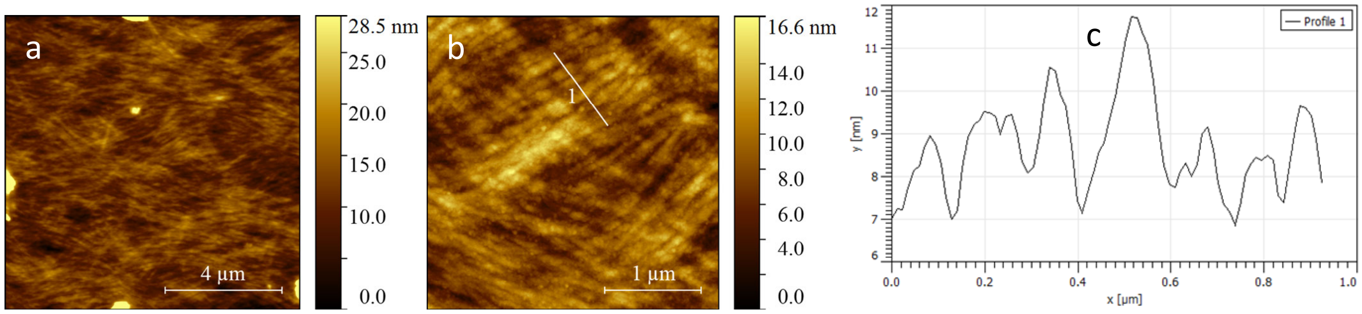

Figure S17. AFM images of G4-CD_5: (a) scale bar – 4 µm; (b) scale bar – 1 µm; (c) Z-profiles along the lines marked on the images.

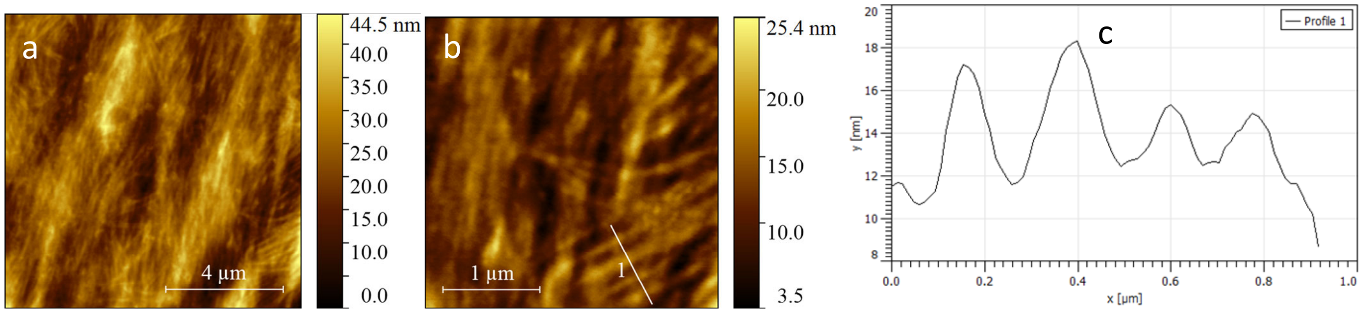

Figure S18. AFM images of G4-CD_6: (a) scale bar – 4 µm; (b) scale bar – 1 µm; (c) Z-profiles along the lines marked on the images.

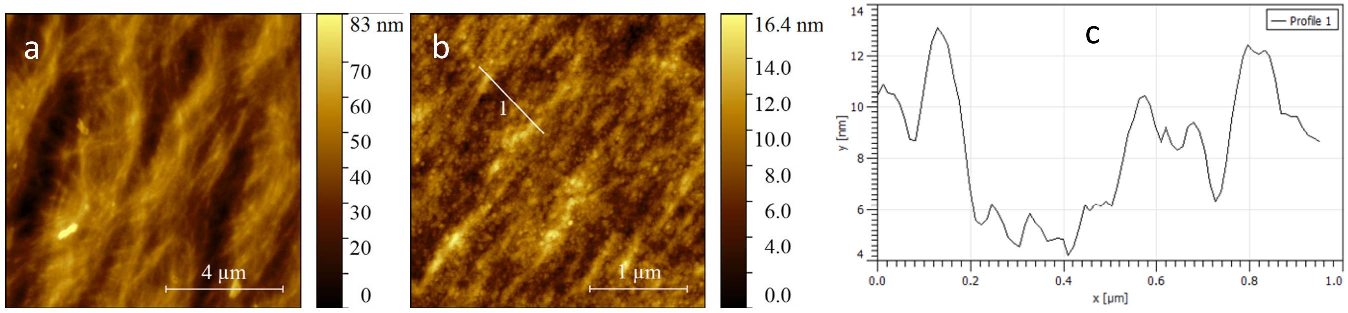

Figure S19. AFM images of G4-CD_7: (a) scale bar – 4 µm; (b) scale bar – 1 µm; (c) Z-profiles along the lines marked on the images.

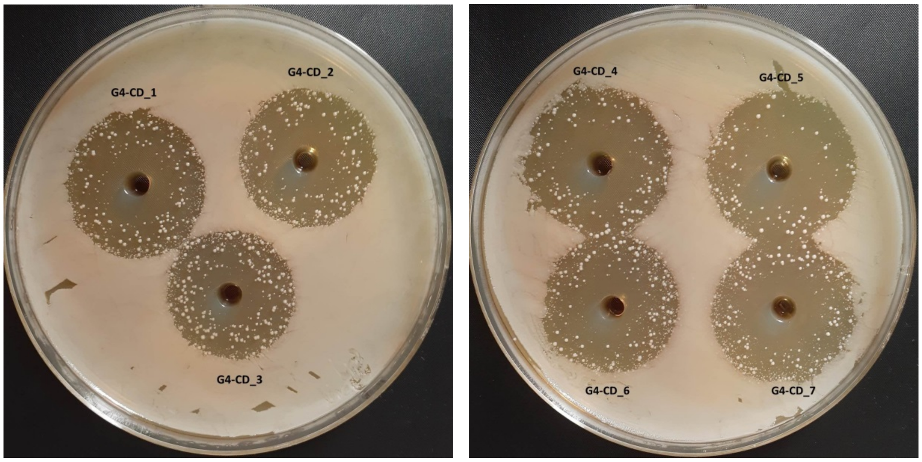

Figure S20. Antibacterial activity of the G4-CD_1-7 hydrogels against *S. aureus*.

Table S1. Antimicrobial activity of the G4-CD_1-7 hydrogels against the references strains.

**Strains**	**Inhibition zone (mm)**						
	**G4-CD_1**	**G4-CD_2**	**G4-CD_3**	**G4-CD_4**	**G4-CD_5**	**G4-CD_6**	**G4-CD_7**
S. aureus	30.30±1.50	30.80±0.80	29.21±0.37	32.99±0.03	33.21±0.71	31.95±0.19	32.13±0.92
E. coli	-	-	-	-	-	-	-
E. faecalis	-	-	-	-	-	-	-
K. pneumonie	-	-	-	-	-	-	-
S. typhimurium	-	-	-	-	-	-	-
C. albicans	-	-	-	-	-	-	-
C. glabrata	-	-	-	-	-	-	-
A. brasiliensis	-	-	-	-	-	-	-
